# Carriage of *Streptococcus pneumoniae* and Other Respiratory Bacterial Pathogens in Low and Lower-Middle Income Countries: A Systematic Review and Meta-Analysis

**DOI:** 10.1371/journal.pone.0103293

**Published:** 2014-08-01

**Authors:** Richard A. Adegbola, Rodrigo DeAntonio, Philip C. Hill, Anna Roca, Effua Usuf, Bernard Hoet, Brian M. Greenwood

**Affiliations:** 1 GlaxoSmithKline Vaccines, Wavre, Belgium; 2 Medical Research Council Unit, Banjul, The Gambia; 3 Centre for International Health, School of Medicine, University of Otago, Dunedin, New Zealand; 4 Faculty of Infectious & Tropical Diseases, London School of Hygiene and Tropical Medicine, London, United Kingdom; Wake Forest University School of Medicine, United States of America

## Abstract

**Background:**

Infection with *Streptococcus pneumoniae* is a major cause of childhood morbidity and mortality worldwide, especially in low income countries where pneumococcal conjugate vaccines (PCVs) are still underused. In countries where PCVs have been introduced, much of their efficacy has resulted from their impact on nasopharyngeal carriage in vaccinated children. Understanding the epidemiology of carriage for *S. pneumoniae* and other common respiratory bacteria in developing countries is crucial for implementing appropriate vaccination strategies and evaluating their impact.

**Methods and Findings:**

We have systematically reviewed published studies reporting nasopharyngeal or oropharyngeal carriage of *S. pneumoniae, Haemophilus influenzae*, *Moraxella catarrhalis*, *Staphylococcus aureus*, and *Neisseria meningitidis* in children and adults in low and lower-middle income countries. Studies reporting pneumococcal carriage for healthy children <5 years of age were selected for a meta-analysis. The prevalences of carriage for *S. pneumoniae, H. influenzae*, and *M. catarrhalis* were generally higher in low income than in lower-middle income countries and were higher in young children than in adults. The prevalence of *S. aureus* was high in neonates. Meta-analysis of data from young children before the introduction of PCVs showed a pooled prevalence estimate of 64.8% (95% confidence interval, 49.8%–76.1%) in low income countries and 47.8% (95% confidence interval, 44.7%–50.8%) in lower-middle income countries. The most frequent serotypes were 6A, 6B, 19A, 19F, and 23F.

**Conclusions:**

In low and lower-middle income countries, pneumococcal carriage is frequent, especially in children, and the spectrum of serotypes is wide. However, because data are limited, additional studies are needed to adequately assess the impact of PCV introduction on carriage of respiratory bacteria in these countries.

## Introduction

*Streptococcus pneumoniae* (the pneumococcus) is a major cause of invasive diseases and respiratory tract infections and caused approximately 500,000 deaths in children <5 years of age in 2008, mostly in low income countries [1], [2]. Asymptomatic nasopharyngeal carriage, which plays an essential role in the transmission of *S. pneumoniae*, usually precedes invasive pneumococcal disease (IPD) [3].

Since 2000, the introduction of the 7-valent pneumococcal conjugate vaccine (PCV-7,) and second generation PCVs has significantly reduced the burden of IPD in vaccinated children [4]–[11]. PCVs have also reduced IPD in unvaccinated children and adults [5], [8], [10], [12]. The indirect (or herd) effects of PCVs against IPD are driven primarily by their impact on carriage of vaccine-type (VT) pneumococci in vaccinated children, preventing transmission to unvaccinated contacts [13]–[15].

Reduction in carriage of VT pneumococci due to PCV vaccination is often accompanied by an increase in the carriage of non-vaccine-type (NVT) pneumococci and, to a lesser extent, an increase in the incidence of IPD caused by NVT pneumococci [7], [8], [14], [16], [17]. However, the overall benefit of PCV-7 vaccination has not been substantially affected by replacement disease [7], [8], [18], [19] except in Alaska and possibly the United Kingdom [5], [20].

By reducing nasopharyngeal carriage of VT pneumococci, PCVs may also create ecological niches for colonization of alternative respiratory pathogens such as *Staphylococcus aureus*, *Haemophilus influenzae*, and *Moraxella catarrhalis* [21], bacteria that are frequently carried by children and are important causes of disease worldwide [22]–[24]. Introducing PCVs may also have modified carriage of *Neisseria meningitidis* in children, although the prevalence of meningococcal carriage is usually higher in adolescents and young adults [25]. Vaccination with PCV-7 has been associated with a shift in the etiology of acute otitis media (AOM) so that non-typeable *H. influenzae* has become the leading cause of AOM in place of *S. pneumoniae* in several studies [26], [27]. In addition, some studies have shown that carriage of *S. pneumoniae* is inversely related to carriage of *S. aureus* in healthy children [21], [28], [29]. Clarifying the interactions between these different pathogens is essential to assess overall impact on conjugate vaccines. *H. influenzae* type b (Hib) and *N. meningitidis* conjugate vaccines are also available and being introduced in low income countries, which may further influence global patterns of bacterial carriage [30].

Because the burden of IPD is high in young children in developing countries, second generation PCVs including more pneumococcal serotypes are being rapidly introduced into the Expanded Programme on Immunization of low and lower-middle income countries [2], [31]. Given the impact of PCV-7 childhood vaccination on carriage and the subsequent indirect benefits seen in industrialized countries, understanding the dynamics of pharyngeal carriage of pneumococci in developing countries is crucial to predict the potential public health implications of routine PCV use in these settings. Therefore, we have conducted a systematic literature review to evaluate nasopharyngeal and oropharyngeal carriage prevalence of *S. pneumoniae* in children and adults from low and lower-middle income countries, with a focus on children <5 years of age. We have also analyzed the prevalence of carriage of other common pathogenic respiratory bacteria that could be affected by the introduction of PCVs.

## Methods

### Search strategy and selection criteria

We systematically reviewed the literature on the carriage of *S. pneumoniae*, *H*. *influenzae*, *M. catarrhalis*, *S. aureus*, and *N. meningitidis* in low and lower-middle income countries, as defined by the World Bank (Text S1) [32]. We searched PubMed for articles published in English between January 1, 1990 and October 23, 2012 using a combination of search strings for microbiological agents, colonization and carriage, and low income and lower-middle income countries (Text S1). Websites of the World Health Organization (www.who.int), the United Nations International Children's Emergency Fund (www.unicef.org), and Google (www.google.com) were searched for additional data. The review is reported according to the PRISMA statement (Table S2) [33].

Two reviewers independently screened the titles and abstracts of all retrieved articles. Disagreements between the two reviewers were resolved by discussion. Articles that did not contain relevant information were excluded. In a second step, the full text of the selected articles was assessed for eligibility using pre-defined inclusion and exclusion criteria (Protocol S1).

All articles relevant to the objectives of the review and that reported prevalences of nasopharyngeal or oropharyngeal carriage of *S. pneumoniae*, *H*. *influenzae*, *M. catarrhalis*, *S. aureus*, or *N. meningitidis* in children or adults of all ages were included. Articles were excluded if they were letters (except those reporting results from studies that had a reasonable sample size and interpretable results), editorials, comments, or diagnostic articles; lacked methodology (e.g. no inclusion or exclusion criteria, setting not clearly described, or were biased); the location from which the swab was collected, the culture plate, or the method for identifying pathogens was unclear; reported phase II trials; reported randomized controlled trials with no usable intervention or placebo group (if only the placebo group was usable, the article was included but only the results of the placebo arm were considered); reported studies with a sample size <50 (except for articles on *M. catarrhalis*); were reviews that included only articles already covered by the current search.

### Assessment of study quality

Reviewers assessed the quality of the studies reviewed by adapting the criteria of the Coordination of Cancer Clinical Practice Guidelines [34] for epidemiological studies. Specifically, the quality of the following were scored as - -, -, 0, +, or ++: study population clearly described and representative of the source population; outcome measures described; results applicable to the subject group targeted in the review; study design and setting described; main potential confounders identified and taken into account in the design and analysis; confidence intervals provided; type of sample collected and the typing method described clearly. This checklist was not designed to calculate a global quality score for each study. In case of doubt whether the quality of a study was sufficient for inclusion, the reviewer discussed the article with a second reviewer.

### Data extraction and analysis

An EndNote (Thomson Reuters, New York, NY) database containing all the included and excluded articles was created. Extracted data included country, setting, study years, study design, population studied, type of swab obtained, culture and identification methods, number of subjects, number of swabs, definition of prevalence, and prevalence of carriage.

Prevalence of carriage (with 95% confidence interval [CI] if available) was reported for each pathogen for healthy, immunocompromised, and ill populations. Ill populations were defined as subjects with IPD, AOM, sinusitis, or respiratory tract infection (RTI). When subjects were swabbed once, point prevalence was defined as the number of positives per total samples or subjects. When subjects were swabbed several times, average prevalence was defined as the total number of positive samples divided by the total number of samples collected, without taking into account the number of samples taken per subject or the duration of the study. Period prevalence was defined as the percentage of subjects who were positive at some point during the study period. The serotype distribution (expressed as percentage of subjects or isolates) and the bacterial density (expressed as arbitrary units [35] or bacterial load calculated as genome equivalent/mL sample secretion [36]) were also analyzed when data were available.

### Meta-analysis

Studies with a similar study design that reported *S. pneumoniae* carriage for healthy children <5 years of age were selected for a meta-analysis. Studies were included in the meta-analysis if the articles reported point prevalence estimates (with available number of subjects positive for carriage and sample size in the target age group) and if they included at least 100 subjects. Pooled prevalence estimates of carriage were computed when possible using the random-effects model of DerSimonian-Laird [37]. Heterogeneity between studies was assessed using the I^2^ statistic. Stata version 11 (StataCorp, College Station, TX) and Metalight (Metalight Systems, Round Rock, TX) were used for all analyses.

## Results

### Results of the literature search

**Figure 1:**
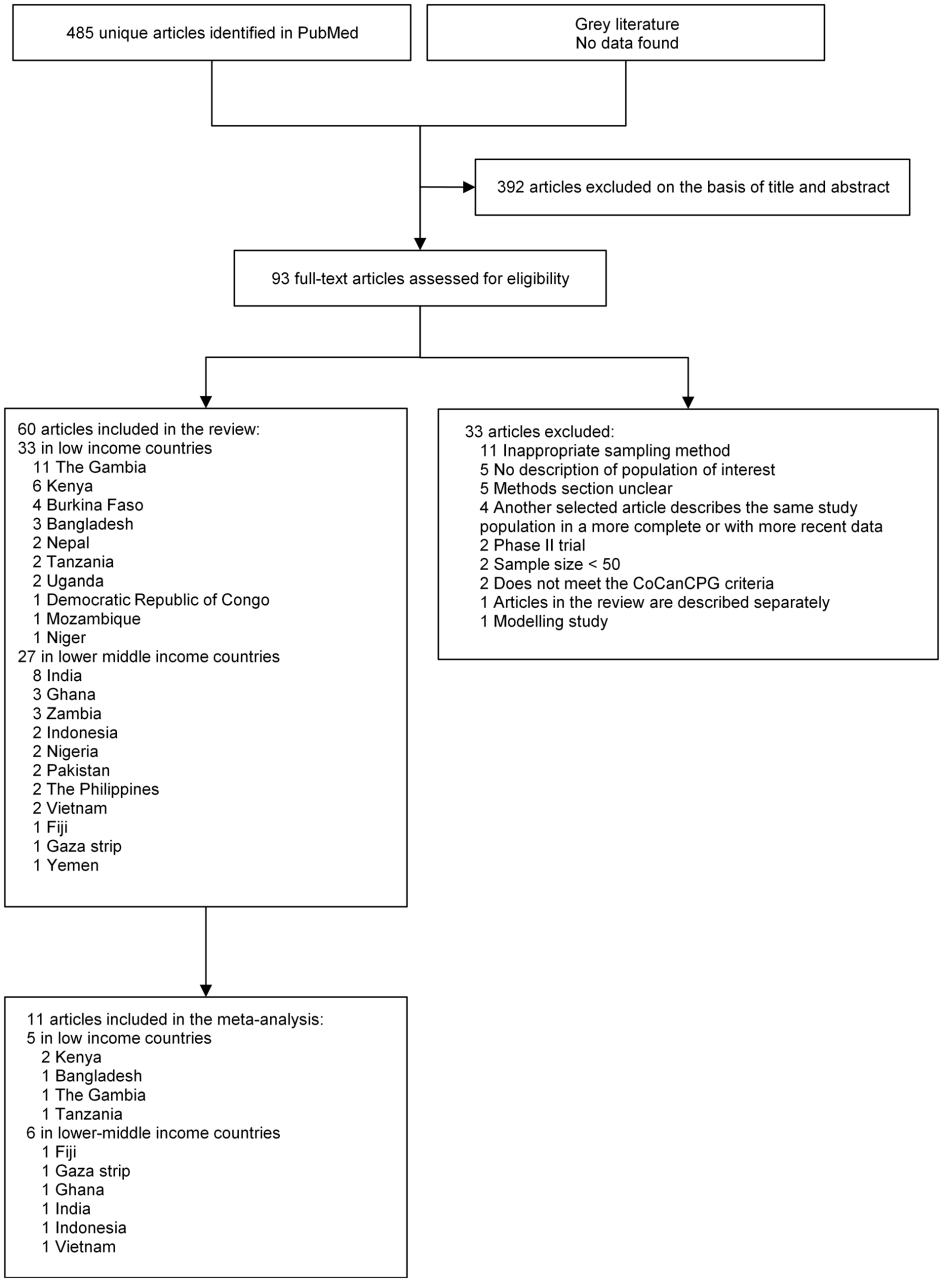
Flowchart of study selection. CoCanCPG, Coordination of Cancer Clinical Practice Guidelines.

We identified 485 articles related to carriage of potentially pathogenic bacteria from PubMed; 93 were retrieved for assessment of the full text (Figure 1). Sixty of these articles were included in the systematic review, 33 of which were from low income countries and 27 from lower-middle income countries. These studies were most frequently performed in Africa (N = 38) and Asia (N = 21). One article was from Oceania. A total of 44 articles reported carriage results for *S*. *pneumoniae*, 12 for *H. influenzae*, three for *M. catarrhalis*, four for *S. aureus*, and nine for *N. meningitidis*. Eight articles reported prevalence of carriage for more than one pathogen.

### Carriage of *Streptococcus pneumoniae*

Thirty-two of the 44 studies that reported prevalence of *S. pneumoniae* carriage were conducted in healthy populations, eight in immunocompromised populations, and six in populations with IPD, AOM, sinusitis, or RTI (Table 1, Table S2). Three articles reported prevalence of carriage in both healthy and ill populations [36], [38], [39].

**Table 1:** Summary table of studies reporting carriage of *Streptococcus pneumoniae*.

Reference		Study period	Country	Sample size	Prevalence	Age group	Prevalence of carriage, % (95% CI)
***Low income countries***							
**Healthy population**							
[65]	Saha et al. 2003	1999–2000	Bangladesh	2839 children	Point	Total	46
[49]	Granat et al. 2007	2000–2001	Bangladesh	98 families with 99 new-borns	Average	4–12 months	49.3
						1–4 years	50.9
						5–9 years	41.5
						10–18 years	32.4
						Mothers	7.3
						Other adults	8.2
[116]	Coles et al. 2011	2005–2007	Bangladesh	225 children	Point	12 weeks	72.9
[42]	Cheung et al. 2009	2003 and 2003–2004	The Gambia	2342 children and 675 of their younger siblings	Point and average	Median age: 12 months. Median age siblings: 3	86.2^a^
[53]	Roca et al. 2011	2003–2008	The Gambia	2094 individuals pre-vaccination	Point	2–<5 years	93.4^a^
						5–<15 years	86.3
						≥15 years	60.6
[43]	Kwambana et al. 2011	NR	The Gambia	30 infants	Average	0–12 months	78 (76–83)
[54]	Hill et al. 2010	NR	The Gambia	158 individuals	Period	Children	97 (over study period)
					Period	Adults	85 (over study period)
[50]	Hill et al. 2008	NR	The Gambia	236 infants	Period	0–11 months	20–90 (100 over study period)
[57]	Hill et al. 2006	NR	The Gambia	2972 individuals	Point	Median age: 15 years	72
[39]	Lloyd-Evans et al. 1996	1989–1991	The Gambia	113 children	Point	<5 years	76.1
[55]	Abdullahi et al. 2008	2004	Kenya	450 individuals	Average	0–4 years	57
						5–9 years	41 (32–51)
						10–85 years	6.4
[52]	Abdullahi et al. 2012	2006–2009	Kenya	2840 children	Point	3–59 months	65.8
[51]	Tigoi et al. 2012	2006–2009	Kenya	1404 children, 1372 mothers, 221 fathers, and 1412 siblings	Average	Mean: 2.1 days. Mean age family members: NR	63.2^b^
[64]	Valles et al. 2006	2003	Mozambique	285 children	Point	<5 years	87
[38]	Coles et al. 2008	2003–2005	Nepal	197 children	Point	1–36 months	78.7^a^
[63]	Moyo et al. 2012	2010	Tanzania	300 children	Point	<5 years	35
**Immunocompromised population**							
[117]	Rusen et al. 1997	1990	Kenya	26 children with HIV	Point	<5 years	86
[58]	Abdullahi et al. 2012	2006–2008	Kenya	99 children with HIV	Point	3–59 months	76 (66–84)
[41]	Anthony et al. 2012	2008	Tanzania	142 children with HIV	Point	1–4 years	88
						5–9 years	77
						10–14 years	76
[59]	Kateete et al. 2012	2001–2002	Uganda	81 children with homozygote sickle cell disease	Point	8 months–6 years	33
[40]	Blossom et al. 2006	2004–2005	Uganda	600 individuals with HIV	Point	Adults (mean age 38.15 years)	18
**Sick population**							
[39]	Lloyd-Evans et al. 1996	1989–1991	The Gambia	1152 children: 1071 sick (clinical diagnosis of pneumonia, meningitis, septicemia, or other serious bacterial infection) and 81 with IPD	Point	<5 years	Sick: 85.1. IPD: 90.1
[38]	Coles et al. 2008	2003–2005	Nepal	197 children with ALRI	Point	1–36 months	80.2^a^
***Lower-middle income countries***							
**Healthy population**							
[68]	Russell et al. 2006	2003–2004	Fiji	774 children	Point	3–13 months	44.3
[56]	Regev-Yochay et al. 2012	2009	Gaza strip	379 children	Point	3 weeks–5.5 years	50
				379 parents		NR	8
[66]	Denno et al. 2002	1996	Ghana	311 children	Point	6–12 months	51.4
[118]	Donkor et al. 2010	2006–2007	Ghana	124 children	Point	<13 years	15.3
[119]	Coles et al. 2001	1998–1999	India	225 infants	Point	2 months	54.2
						4 months	67.9
						6 months	69.8
[75]	Das et al. 2002	2000–2001	India	566 children	Point	5–12 years	29.1
[120]	Devi et al. 2012	2009–2010	India	811 children	Point	0–14 years	12.8
[67]	Rupa et al. 2012	2009–2010	India	210 children	Point	0–1 years	Maximum: 46.3
[121]	Wattal et al. 2007	NR	India	200 children	Point	3 months–3 years	6.5
[69]	Soewignjo et al. 2001	1997	Indonesia	484 children	Point	0–25 months	48 (42–54)^c^
[122]	Adetifa et al. 2012	NR	Nigeria	1005 individuals	Point	All ages	52.5 (49.4–55.7)
[36]	Vu et al. 2011	2007–2008	Vietnam	350 children	Point	<5 years	50.3
[123]	Gill et al. 2008	2003–2005	Zambia	132 children born to HIV+ mothers and 128 children born to HIV− mothers	Average	6 weeks	25.8
**Immunocompromised population**							
[70]	Bhattacharya et al. 2012	2008–2009	India	148 children with HIV	Point	1–16 years	28
[71]	Mwenya et al. 2010	2002–2003	Zambia	439 children with HIV	Point	6 months–14 years	51
[124]	Gill et al. 2008	2003–2005	Zambia	132 women with HIV	Average	Mean: 25.9 years	11.4
**Sick population**							
[62]	Mastro et al. 1993	1989–1990	Pakistan	601 children with ARI	Point	Mean:14.5 months	64.4
[60]	Lankinen et al. 1994	1984	The Philippines	318 children with ALRI	Point	<5 years	51
[61]	Lupisan et al. 2000	1994	The Philippines	956 children with severe pneumonia, suspected meningitis, or clinical suspicion of sepsis.	Point	0–59 months	27.9
[36]	Vu et al. 2011	2007–2008	Vietnam	274 children with radiologically confirmed pneumonia	Point	<5 years	38.7
				276 children with other LRTI			43.3

ALRI, acute lower respiratory infection; ARI, acute respiratory infection; HIV, human immunodeficiency virus; IPD, invasive pneumococcal disease; LRTI, lower respiratory tract infection; NR, not reported.

a Carriage rate is from the control group.

b Overall carriage rate. Children, mothers, fathers, and siblings combined.

c Age- and population-weighted carriage rate, adjusted for design effect.

One study included oropharyngeal rather than nasopharyngeal samples [40] and four studies used polymerase chain reaction (PCR) rather than conventional microbiology to detect carriage [36], [41]–[43]. Fourteen studies mentioned that sampling, storage, and culture procedures followed the WHO guidelines for detecting carriage of *S. pneumoniae* [44], [45]. Calcium alginate was the most frequent type of swab used (17/44 studies; 38.6%). Four articles did not report overall prevalence of pneumococcal carriage and are therefore not listed in Table 1, although they were included in the review for other outcomes. Three of these articles reported prevalence of pneumococcal carriage only by serotype [46]–[48], and one reported an overall carriage rate that had been published previously but reported a secondary analysis of bacterial density and impact of vaccination [35]. All the studies were performed before the introduction of PCV into the national childhood immunization program.

The prevalences of *S. pneumoniae* carriage in healthy children <5 years of age ranged from 20% to 93.4% in low income countries and were generally higher than reported in lower-middle income countries (range, 6.5%–69.8%). Two longitudinal studies undertaken in The Gambia and Bangladesh reported that all children carried *S. pneumoniae* at least once during their first year of life [49], [50]. In The Gambia, carriage rates increased from 20% within a week of birth to 80%–90% between 3 and 11 months of age [50]. A similar result was found using PCR [43]. The median age at first acquisition of *S. pneumoniae* was 24 days (95% CI, 21–28) in The Gambia and 38.5 days in Kenya [50], [51]. In Kenya, the acquisition rate of pneumococcal carriage was 0.0189 acquisitions/day (95% CI, 0.0177–0.0202 acquisitions/day) in newborns and 0.061 acquisitions/day (95% CI, 0.055–0.067) in children <5 years of age [51], [52]. In all the studies that included both children and adults, the prevalence of carriage was higher in children than in adults, with the highest rates found in children <5 years of age [49], [53]–[56]. The highest prevalence of carriage was in The Gambia [39], [42], [43], [50], [53], [54], [57]. No major difference in prevalence was seen between rural or urban settings in lower-middle income countries, but data were not available for urban areas in the low income countries.

All but one study that reported the prevalence of pneumococcal carriage in immunocompromised populations were conducted in Human Immunodeficiency Virus (HIV)-infected populations. *S. pneumoniae* carriage prevalence in HIV-infected subjects decreased with age (76%–88% in children <5 years of age vs. 11.4%–18% in adults). In Kenya, the prevalence of carriage in HIV-infected children 3–59 months of age was higher than in those uninfected (76% [95% CI, 66%–84%] vs. 65.8% [95% CI, 64.0%–67.5%], p = 0.04) [52], [58]. One study, from a low income country (Uganda), reported a 33% prevalence in children aged 8 months–6 years with homozygote sickle cell disease, a prevalence which was lower than in HIV-infected children in the same age category [59].

Six studies reported prevalence of *S. pneumoniae* carriage in ill subjects, all in children <5 years of age. Prevalence rates ranged from 80.2% to 90.1% in low income countries and from 27.9% to 64.4% in lower-middle income countries [36], [38], [39], [60]–[62]. The prevalence of carriage was generally lower in children with radiologically confirmed pneumonia (RCP, 38.7%) than in children with IPD (90.1%) or RTIs (43.3%–80.2%).

#### Serotype distribution

Overall serotype distributions were reported for 33 studies, 22 of which reported details of the particular serotypes or serogroups detected (Table S3). Overall, a wide spectrum of serotypes was detected, with up to 74 different serotypes isolated in one study [57]. Serotypes 6A, 6B, 19A, 19F, and 23F were the serotypes most commonly isolated. Serotypes 14 and 11A were also frequently isolated. A longitudinal study in The Gambia showed that serotypes 6B, 19F, 23F, and 6A were the most commonly isolated pneumococci at first acquisition, whereas serotypes 6B, 19F, 14, and 6A were the most common isolated in subsequent episodes of carriage [50]. In addition, NVT were acquired faster (median age at acquisition, 152 days vs. 174; p = 0.004) and had a shorter duration of carriage (median duration, 43 vs. 70 days; p<0.001) than PCV7-serotypes [50]. Between 36% and 56% of the pneumococcal isolates detected in unvaccinated healthy children <5 years of age in Africa were of PCV-7 serotypes [50], [51], [57], [63], [64], whereas the proportions were 37%–56% for PHiD-CV (10-valent pneumococcal *Haemophilus influenzae* protein D conjugate vaccine) and 50%–64% for PCV-13 (13-valent pneumococcal conjugate vaccine) [51], [63]. Non-typeable *S. pneumoniae* represented between 0.7% [55] and 20.6% [60] of the isolates. The highest percentage of non-typeable *S. pneumoniae* was in Filipino children with acute lower respiratory tract infection [60].

#### Meta-analysis of pneumococcal carriage

**Figure 2:**
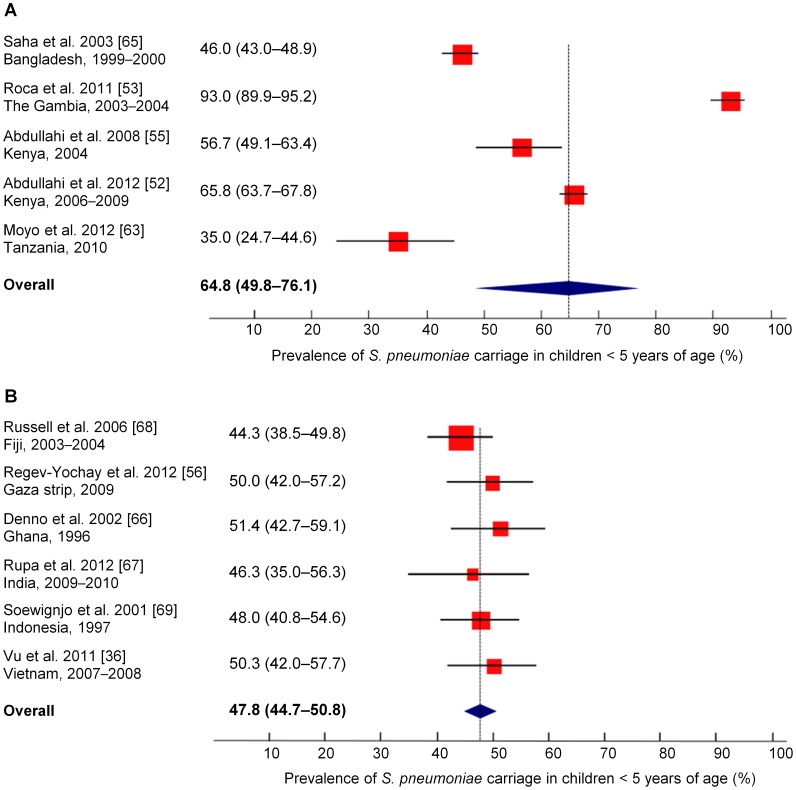
Meta-analysis of *S. pneumoniae* carriage in healthy children under 5 years of age. (A) Low income countries. (B) Lower-middle income countries. The point prevalence estimate for each study is represented by a square. The 95% confidence interval (CI) for each study is represented by a horizontal line crossing the square. The size of the square corresponds to the weight of the study in the meta-analysis. All data were obtained before the introduction of the 7-valent pneumococcal conjugate vaccine.

The pooled prevalence for carriage o*f S. pneumoniae* in healthy children <5 years of age was 64.8% (95% CI, 49.8%–76.1%) for the five populations from low income countries [52], [53], [55], [63], [65] and 47.8% (95% CI, 44.7%–50.8%) for the six populations from lower-middle income countries (Figure 2) [36], [56], [66]–[69]. However, studies were highly heterogeneous in the low income countries (I^2^ = 98%), in contrast to those in the lower-middle income countries (I^2^ = 0%).

### Carriage of *Haemophilus influenzae*

The highest prevalence of *H. influenzae* carriage detected in a healthy population (70%) was recorded in a longitudinal study in infants in a rural setting in The Gambia (Table 2, Table S4) [43]. The study, which used PCR to detect respiratory pathogens, reported that carriage of *H. influenzae* was infrequent at birth (<20% within the first week of life) and increased steadily with age (>90% at 19 weeks), with a pattern similar to that of *S. pneumoniae* [43]. Another study in a post-Hib vaccination setting reported that *H. influenzae* carriage rates were lower in children aged ≥10 years and adults (1.1%–3.9%) than in children <10 years (21.0%–29.0%) [55]. The prevalence of *H. influenzae* carriage in HIV-infected children was reported for India (24%) [70] and Zambia (29%) [71], two lower-middle income countries. The prevalence of *H. influenzae* carriage in ill children from three lower-middle income countries ranged between 15.2% and 53.5% [36], [61], [62]. PCR generally yielded higher rates of *H. influenzae* carriage than microbiology in both healthy (31.4%–70% vs. 1.1%–28.6%) and ill (50%–53.5% vs. 15.2%–36.6%) subjects.

**Table 2:** Summary table of studies reporting carriage of *Haemophilus influenzae*.

Reference		Study period	Country	Sample size	Prevalence	Age group	Prevalence of carriage, % (95% CI)
***Low income countries***							
**Healthy population**							
[43]	Kwambana et al. 2011	NR	The Gambia	30 infants	Average	0–12 months	70 (65–74)
[72]	Adegbola et al. 1998	NR	The Gambia	3986 children	Point	1–2 years	Urban: 5.6^a^ Rural: 10.9
[55]	Abdullahi et al. 2008	2004^b^	Kenya	450 individuals	Point	0–4 years	26.0
						5–9 years	24.0 (16–33)
						10–85 years	3.0
[74]	Williams et al. 2011	2007	Nepal	2195 children	Point	0–13 years	5.0 (3.9–6.4)
**Immunocompromised population**							
No data found							
**Sick population**							
No data found							
***Lower-middle income countries***							
**Healthy population**							
[75]	Das et al. 2002	2000–2001	India	566 children	Point	5–12 years	28.6
[76]	Sekhar et al. 2009	2005–2006	India	1000 children	Point	<2 years	11.2
[73]	Gessner et al. 1998	NR	Indonesia	484 children	Point	0–24 months	4.6 (3.7–5.5)^c^
[36]	Vu et al. 2011	2007–2008	Vietnam	350 children	Point	<5 years	31.4
**Immunocompromised population**							
[70]	Bhattacharya et al. 2012	2008–2009	India	148 children with HIV	Point	1–16 years	24
[71]	Mwenya et al. 2010	2002–2003	Zambia	439 children with HIV	Point	6 months–14 years	29
**Sick population**							
[62]	Mastro et al. 1993	1989–1990	Pakistan	601 children with ARI	Point	Mean:14.5 months	36.6
[61]	Lupisan et al. 2000	1994	The Philippines	956 children with severe pneumonia, suspected meningitis, or clinical suspicion of sepsis	Point	0–59 months	15.2
[36]	Vu et al. 2011	2007–2008	Vietnam	274 children with radiologically confirmed pneumonia	Point	<5 years	50
				276 children with other LRTI			53.5

ARI, acute respiratory infection; HIV, human immunodeficiency virus; LRTI, lower respiratory tract infection; NR, not reported.

a All carriage rates are from the control group.

b Post-vaccination data.

c Age- and population-weighted carriage rate, adjusted for design effect.

Nine studies reported distributions for individual *H. influenzae* serotypes, including one conducted after introduction of the Hib vaccine [55]. Three focused on carriage of Hib [72]–[74]. The prevalence of Hib carriage was approximately 5% in these studies, with the highest prevalence (10.9%) in rural children [72]–[74]. The percentage of *H. influenzae* isolates that belonged to serotype b was 0.7%–69% in healthy children [43], [55], [73]–[76], 0.9%–18% in sick children [61], [62], [77], and 49% in immunocompromised children [70]. Non-typeable *H. influenzae* were isolated frequently in studies in which serotyping of *H. influenzae* was undertaken (31%–96.4% of all isolates) [55], [62], [76], except for one study in Nepalese children that reported a prevalence of non-typeable *H. influenzae* of only 1.5% based on oropharyngeal swabs [74].

### Carriage of *Moraxella catarrhalis*

Only three studies reported prevalence of *M. catarrhalis* carriage (Table 3, Table S5) [36], [43], [78]. The highest carriage rate (71%) in healthy subjects was found in a longitudinal study in infants in The Gambia [43] which used PCR. In a study of *M. catarrhalis* carriage detected by microbiological methods in both nasopharyngeal and oropharyngeal samples, carriage prevalence was higher in children (31.4%–38.5%) than in adults (11.7%) [78]. Two of these studies also reported the prevalence of carriage of *M. catarrhalis* in an ill population [36], [78]. In Yemen, the prevalence in children with sinusitis was lower than in children with otitis media or tonsillitis/pharyngitis [78]. However, this study included only 64 ill children. In Vietnam, the prevalence of *M. catarrhalis* carriage in children <5 years of age with RCP was 28.1% compared to a prevalence of 42.2% in children with other lower RTIs (P = 0.001) [36].

**Table 3:** Summary table of studies reporting carriage of *Moraxella catarrhalis*.

Reference		Study period	Country	Sample size	Prevalence	Age group	Prevalence of carriage, % (95% CI)
***Low income countries***							
**Healthy population**							
[43]	Kwambana et al. 2011	NR	The Gambia	30 infants	Average	0–12 months	71 (67–75)
**Immunocompromised population**							
No data found							
**Sick population**							
No data found							
***Lower-middle income countries***							
**Healthy population**							
[36]	Vu et al. 2011	2007–2008	Vietnam	350 children	Point	<5 years	58
[78]	Sehgal et al. 1994	1992–1993	Yemen	35 children	Point	<3 years	31.4
				96 school children		4–12 years	38.5
				120 university students and staff		20–40 years	11.7
**Immunocompromised population**							
No data found							
**Sick population**							
[36]	Vu et al. 2011	2007–2008	Vietnam	274 children with radiologically confirmed pneumonia	Point	<5 years	28.1
				276 children with other LRTI			42.2
[78]	Sehgal et al. 1994	1992–1993	Yemen	64 children with respiratory infections:	Point	3–12 years	21.9
				- OM			26.9
				- Sinusitis			9.1
				- Tonsillitis/Pharyngitis			22.2

LRTI, lower respiratory tract infection; NR, not reported; OM, otitis media.

### Carriage of *Staphylococcus aureus*

Four studies reported prevalence of *S. aureus* carriage (Table 4, Table S6) [43], [70], [79], [80]. All included nasopharyngeal samples. A longitudinal study in The Gambia found an average prevalence of nasopharyngeal *S. aureus* carriage of 20% in healthy infants [43]. The prevalence of carriage was highest in neonates and decreased with age (from 50% to 10%), in contrast to the prevalence of carriage of *S. pneumoniae*, *H. influenzae*, and *M. catarrhalis*, which increased with age during the first year of life [43]. In a second study in Pakistan, 14.8% of subjects of all ages carried *S. aureus*, without a statistically significant difference between age groups [80]. Similar prevalences of carriage of *S. aureus* were reported for two immunocompromised populations, HIV-infected adults in a low income country (27%) and HIV-infected children ≤16 years in a lower-middle income country (26%) [70], [79]. The prevalence of *S. aureus* carriage in an ill population (with IPD, AOM, RTI, or sinusitis) has not been reported.

**Table 4:** Summary table of studies reporting carriage of *Staphylococcus aureus*.

Reference		Study period	Country	Sample size	Prevalence	Age group	Prevalence of carriage, % (95% CI)
***Low income countries***							
**Healthy population**							
[43]	Kwambana et al. 2011	NR	The Gambia	30 infants	Average	0–12 months	20 (16–24)
**Immunocompromised population**							
[79]	Amir et al. 1995	1992	Kenya	264 adults with HIV	Point	NR	27
**Sick population**							
No data found							
***Lower-middle income countries***							
**Healthy population**							
[80]	Anwar et al. 2004	2002–2003	Pakistan	1660 individuals	Point	All ages	14.8
**Immunocompromised population**							
[70]	Bhattacharya et al. 2012	2008–2009	India	148 children with HIV	Point	1–16 years	26
**Sick population**							
No data found							

HIV, human immunodeficiency virus; NR, not reported.

### Carriage of *Neisseria meningitidis*

Nine studies analyzed prevalence of *N. meningitidis* carriage, all in healthy populations (Table S7). Four studies used oropharyngeal samples, three used nasopharyngeal samples, one used both oropharyngeal and nasopharyngeal samples, and one used pharyngeal samples. Carriage rates ranged from 3.17% to 22% in low income countries [81]–[86] and from 1.64% to 6.2% in lower-middle income countries [87]–[89]. However, most studies included both children and adults, and only one described the prevalence of *N. meningitidis* carriage in young children (0.78% in children aged 3–24 months in western Democratic Republic of Congo) [81], the main target age group of this review. In addition, previous reviews suggest that infants and young children are unlikely to carry *N. meningitidis* [25], [90]. For these reasons, the results of the systematic search for this pathogen are not described further in this review.

### Risk factors for carriage

Twenty-five studies evaluated possible risk factors for carriage of *S. pneumoniae*. Among healthy subjects, seasonality was identified as a risk factor for *S. pneumoniae* carriage in Kenya [51], [55], whereas day care attendance was identified as a risk factor in the Gaza strip [56]. Conflicting results were reported for several risk factors, including age, living in crowded conditions or a rural area, co-carriage with other pathogens, RTIs, or HIV infection. Co-carriage with *H. influenzae* was the only significant risk factor identified in an immunocompromised population [70], whereas an age >2 months and no previous antibiotic use were identified as risk factors for carriage in subjects with IPD, AOM, sinusitis, or RTI [60], [61].

For carriage of *H. influenzae*, risk factors were an age >2 months, co-colonization with *S. pneumoniae*, and the winter or rainy season [55], [76]. Risk factors reported for carriage with *S. aureus* were living in an urban setting and HIV infection [79], [80]. Risk factors for carriage of *M. catarrhalis* were not reported in any study.

### Association between carriage and disease

An association between carriage and disease was analyzed in only two studies. One study, conducted in India, linked *S. pneumoniae* carriage to RTIs [67]. Children with six or more episodes of upper RTI had a higher percentage of pneumococcal-positive swabs than children who had less than three upper RTI episodes (60% vs. 17.2%; P<0.001). In multivariate analysis, significant risk factors for upper RTIs in the first year of life were nasopharyngeal carriage of *S. pneumoniae*, winter season, and a lower socioeconomic status of the parents [67]. The other study, conducted in The Gambia, linked serotype-specific carriage of *S. pneumoniae* to IPD [39]. In 43 of 56 (76.8%) children with IPD, pneumococci isolated from the nasopharynx and from the blood or other sterile site belonged to the same serotype.

### Bacterial density

Only two studies analyzed the density of bacterial carriage [35], [36]. In a group of villages in which only children <30 months of age received PCV-7, the density of pneumococcal carriage (expressed as arbitrary units ranging from 1 to 4) decreased from 2.76 to 1.99 (p = 0.002) in children ≤5 years and from 2.44 to 1.75 (p = 0.001) in individuals >5 years, suggesting naturally acquired immunity [35].

The second study reported the density of *S. pneumoniae*, *H. influenzae*, and *M. catarrhalis* in healthy children, children with RCP, and children with other lower RTIs [36]. The bacterial load of *S. pneumoniae* in the nasopharynx, calculated as genome equivalent/mL sample secretion, was higher in children with RCP (7.8×10^6^/mL) than in children with other lower RTIs (1.3×10^6^/mL; p<0.0001) and healthy children (7.9×10^5^/mL; p<0.0001). *M. catarrhalis* bacterial load was higher in children with RCP (2.5×10^7^/mL) and other lower RTIs (3.3×10^7^/mL) than in healthy children (5.5×10^6^/mL; p<0.0001). *H. influenzae* bacterial load was lower in children with other lower RTIs than in children with RCP (p = 0.003) but there was no significant difference between healthy children and children with RCP [36].

### Impact of vaccination on carriage

Five studies, all in The Gambia, analyzed the impact of pneumococcal vaccination on *S. pneumoniae* carriage rate and serotype distribution (Table S8) [35], [42], [46], [47], [53]. All of these studies were performed before PCV7 was introduced into the Gambian national childhood immunization program in 2009 [91]. Cheung et al. reported similar total carriage rates for children who had received the 9-valent PCV (PCV-9; 87.5%) and those who had received a placebo vaccine (86.2%) [42]. However, compared to placebo-vaccinated children, PCV-9-vaccinated children were less likely to carry a VT pneumococcus (22.6% vs. 40.0%; relative risk  = 0.56; 95% CI, 0.49–0.65) and more likely to carry a NVT pneumococcus (42.7% vs. 26.9%, relative risk  = 1.59; 95% CI, 1.41–1.79). Ota et al. analyzed *S. pneumoniae* carriage rates in 5-, 11-, and 15-month-old children who had received one, two, or three doses of PCV-7 [47]. Carriage rates were similar overall, although carriage rates tended to be higher in children who had received one dose of PCV-7 than in those who had received two or three doses. At 11 months of age, carriage of a VT pneumococcus was more common in children who had received one dose of PCV-7 than in children who had received three doses (20.2% vs. 10.0%, p = 0.005). In a cluster-randomized trial in which children <30 months of age were vaccinated with PCV-7, Roca et al. showed that the overall carriage rate in residents decreased from 71% in the pre-PCV-7 vaccination period to 44% following PCV-7 vaccination [35], [53]. Carriage rates increased with age (age groups: 2–4, 5–14, and ≥15 years), but they did not differ for any age group at any time point between the villages in which all residents received PCV-7 and those in which only children <30 months of age received PCV-7 [53]. The carriage rates of all age groups were lower at the first (4–6 months after vaccination) and second (12 months after vaccination) post-vaccination surveys than at the pre-vaccination survey. However, by the third post-vaccination survey, 22 months after vaccination, carriage rates were higher than at the second survey but still remained below pre-vaccination rates. Finally, Akinsola et al. compared the prevalence of *S. pneumoniae* carriage in children who had received primary vaccination with PCV-9 followed by a booster dose of PCV-7 and in age-matched children who received only a single dose of PCV-7 [46]. They found no significant differences between the carriage rates or serotype distributions of the two groups at any time point during a 16–18 month follow-up after vaccination. In conclusion, all five studies showed that the prevalence of VT carriage decreased after PCV vaccination, although the extent of the decrease varied between studies.

Two studies reported the impact of Hib vaccination on *H. influenzae* carriage. In one study, Hib carriage was 11% in fully vaccinated Gambian children who had received the diphtheria-tetanus-pertussis vaccine alone (control group) compared to only 4.4% for fully vaccinated children who had received diphtheria-tetanus-pertussis and an Hib conjugate vaccine (p<0.001) [72]. The prevalence of Hib carriage in children who had been partially vaccinated (either in the control or the vaccinated groups) was higher than in children who had received full vaccination. In the second study, conducted in Nepal, none of 27 children <13 years of age who had received at least one dose of the Hib vaccine carried Hib [74].

## Discussion

This systematic review of the literature fills an important gap in knowledge on the carriage of five key pathogens in low and lower-middle income countries. The review found that nasopharyngeal carriage was frequent in these countries. The prevalences of carriage for *S. pneumoniae, H. influenzae*, and *M. catarrhalis* were generally higher in low income countries than in lower-middle income countries and were generally higher in children than in adults. The prevalence of *S. aureus* carriage was highest in neonates.

In low and lower-middle income countries, the prevalence of pneumococcal carriage was high in young children (up to 93.4% in children 2–4 years of age). Furthermore, carriage was acquired within the first months of life and persisted in older children and adults, although at lower levels [50], [51], [53], [54]. In contrast, in upper-middle and high income countries, the highest prevalence of pneumococcal nasopharyngeal carriage recorded was 58% (in healthy children <3 years of age) [92]. This difference could be due to the frequent overcrowded living conditions in developing countries. This view is supported by the high prevalence of carriage found among children living in orphanages or attending day-care centers in industrialized countries and among indigenous populations [93]–[96]. Malnutrition, more common in the developing countries, could also favor carriage and disease, as previously suggested [97].

In addition to a high prevalence of *S. pneumoniae* carriage, young children also have a higher pneumococcal density in the nasopharynx than older individuals [35]. This may partly explain why children are more efficient than adults at transmitting *S. pneumoniae*. The lower density in adults may be due to the progressive acquisition of immunity, less frequent close contact between individuals, better hygiene, or a combination of these factors. IPD is probably also a substantial public health problem for adults in many developing countries [98], particularly for countries with a high incidence of HIV [99]–[101], but this has not been well documented. As life expectancy increases in these countries, the proportion of the adult population with risk factors for IPD, such as diabetes, cardiovascular diseases, and chronic RTIs, is increasing [102]. It is hoped that an indirect effect of pediatric pneumococcal vaccination, through reduced carriage of VT pneumococci, will also be seen in these at risk populations, as it has occurred in the US and Europe [5], [8], [13] but this will occur only if the major route of pneumococcal transmission in these countries is from young children to adults. There is currently little information to support this expectation but longitudinal studies in Kenya and rural Gambia have suggested that intra-household transmission is more important than community transmission and that young children commonly introduce *S. pneumoniae* into the household, with subsequent spread to other children and adults [51], [54]. Vaccinations strategies against pneumococcal disease need to target young children, but a strong case can be made for catch-up campaigns in older children in developing countries because children up to nine years of age can effectively transmit *S. pneumoniae* [51].

The review has shown that residents of low and lower-middle income countries carry a wide variety of pneumococcal serotypes. Identifying clear trends in the serotype distributions was challenging due to the disparate geographic regions represented and to large variations in the types of studies reviewed and sets of serotypes analyzed in each study. Despite this, the five most common serotypes reported (6A, 6B, 14, 19F, and 23F) are among the seven serotypes that globally cause most IPD in children worldwide [103]. The two other serotypes, 1 and 5, are rarely isolated in carriage studies, although they have been found in epidemics of invasive pneumococcal disease [3], [104], [105]. All these serotypes are included in the two second generation PCVs – PHiD-CV and PCV-13.

Studies in industrialized countries have established a temporal association between pneumococcal carriage and disease, especially for AOM. We found only limited data on associations between disease and carriage in low and lower-middle income countries, although associations between *S. pneumoniae* and both IPD and RTIs have been described [39], [67].

Few carriage studies have analyzed more than one pathogen and the interactions between them. In The Gambia, approximately 90% of infants co-carried *S. pneumoniae* with at least one other pathogen, most often *H. influenzae* or *M. catarrhalis* [43]. Co-infections with multiple pathogens can subvert mucosal immune responses to carriage in the upper respiratory tract and can alter disease outcome [106]. In contrast, an inverse association has been found between *S. pneumoniae* and *S. aureus* [21], [28], [29], [106]. However, most studies do not consider that risk factors for these infections may be different and that bacterial interactions within the nasopharynx may influence the prevalence of carriage with individual bacteria.

Only five studies included PCV-vaccinated populations and two studies included Hib-vaccinated populations. Results were conflicting for the impact of PCV vaccination on the overall pneumococcal carriage, although all studies showed that the prevalence of VT carriage decreased after PCV vaccination and that the prevalence of Hib carriage decreased after Hib vaccination. A long-term follow-up of the village-randomized study of PCV-7 in The Gambia found that the decrease in VT carriage was sustained four years after vaccination and that carriage of NVT pneumococci did not significantly increase [107]. Also, PCV-7 indirectly reduced carriage of VT pneumococci in unvaccinated infants <8 weeks of age [108]. More data will be available on the long-term impact of PCV immunization as the use of these vaccines continues to increase in lower income countries.

The results of this review need to be interpreted in light of several considerations. *S. pneumoniae* was the only pathogen for which prevalence of carriage was available for all three subpopulations (healthy, immunocompromised, and ill). Heterogeneity between studies may also have hampered direct comparisons. For instance, differences were noted in study design, the definition of prevalence, sampling technique, laboratory methods, the type of sample, and the period between sample collection and vaccination. Notably, not all the studies of *S. pneumoniae* followed the WHO recommendations for measuring nasopharyngeal carriage [44], [45]. Nasopharyngeal and oropharyngeal swabs were used in the studies, both of which differ in sensitivity for detecting respiratory pathogens [109], [110]. Also, some standard methods for bacterial identification do not reliably distinguish *H. influenzae* from *H. haemolyticus*, a respiratory tract commensal [111]. In addition, variation between studies may be due in part to the type of settings where a study was done. Indeed, several studies included children from outpatient clinics, and selection bias is usually higher in these settings than in studies including children from the community. Studies in the low income countries were highly heterogeneous (I^2^ = 98%), so that the pooled estimate for pneumococcal carriage should be interpreted with caution, as it reflects only findings from countries where carriage studies have been published. In contrast, heterogeneity was low between studies from the lower-middle income countries (I^2^ = 0%). We were unable to perform a meta-analysis for *H. influenzae*, *S. aureus*, and *M. catarrhalis* because of the few studies available. Most studies included in this review had a cross-sectional design, which prevents assessing the effect of season on prevalence of carriage. Finally, because seasonality is a risk factor for carriage for *S. pneumoniae* and the other pathogens [51], [55], [76], it might be a confounding factor in comparisons of carriage rates between different studies.

None of the studies included in the review were from Central or South America, the Caribbean, or Europe due to the absence of any publications that met the inclusion criteria. In addition, we did not include upper-middle income countries in the analysis even though some populations within these countries may share characteristics with populations in low or lower-middle income countries. For instance, in one study in South Africa, prevalence of pneumococcal carriage was high (22.5%–61.0%) in children younger than five years of age [112], whereas a study in Costa Rica showed that infants acquire nasopharyngeal carriage of *S. pneumoniae* at a rate (3.1% at one month of age and 19.4% at one year of age) comparable to that seen in developed countries [113].

### Conclusions

With support from the GAVI Alliance, PCVs of higher valencies are being rapidly introduced into the Expanded Programme on Immunization of low and lower-middle income countries [2], [31]. In the large number of countries where large-scale PCV vaccination is being introduced careful surveillance of IPD is needed. However, due to limited resources, population-based IPD surveillance systems similar to those in the industrialized countries will probably not be possible in most low and lower-middle income countries. Because carriage surveys are relatively cheap and possible compared to population-based IPD surveillance, carriage data could be used instead to indirectly monitor vaccine herd effects and serotype replacement after the introduction of PCVs [114] and in the process for future vaccine licensure [115]. However, the relevance of carriage studies for serotype replacement still needs to be properly assessed, because in many countries, although replacement in carriage is complete, the replacement in disease is small [7], [8], [14], [16], [17]. Because the dynamics of carriage within populations are complex, carefully designed multi-year, longitudinal carriage studies will be needed.

Our systematic review found high prevalences of bacterial carriage in children from low and lower-middle income countries. Although data were limited, we expect that the introduction of routine immunization programs against *S. pneumoniae*, *H. influenzae*, and *N. meningitidis* will help reduce the burden of infectious diseases in these countries and that studies of the effects on carriage may provide important data that can be used to understand overall vaccine effectiveness.

## Supporting Information

Table S1 **PRISMA 2009 checklist.**
(DOCX)

Table S2 **Details of studies reporting carriage of** ***Streptococcus pneumoniae*** **.**
(DOCX)

Table S3 **Studies reporting carriage of individual pneumococcal serotypes or serogroups.**
(DOCX)

Table S4 **Details of studies reporting carriage of** ***Haemophilus influenzae*** **.**
(DOCX)

Table S5 **Details of studies reporting carriage of** ***Moraxella catarrhalis*** **.**
(DOCX)

Table S6 **Details of studies reporting carriage of** ***Staphylococcus aureus*** **.**
(DOCX)

Table S7 **Details of studies reporting carriage of** ***Neisseria meningitidis*** **.**
(DOCX)

Table S8 **Impact of vaccination on** ***Streptococcus pneumoniae*** **carriage by age.**
(DOCX)

Text S1 **Supplementary methods.**
(DOCX)

Protocol S1 **Protocol of the systematic review.**
(PDF)
