# Supplementary material for: Carriage of Streptococcus pneumoniae and Other Respiratory Bacterial Pathogens in Low and Lower-Middle Income Countries: A Systematic Review and Meta-Analysis
Source: PLoS One. 2014 Aug 1;9(8):e103293. doi: 10.1371/journal.pone.0103293 (PMC4118866; doi:10.1371/journal.pone.0103293)
Supplement: Table S2 — Details of studies reporting carriage of Streptococcus pneumoniae . (DOCX) [file pone.0103293.s002.docx]

**Table S2.** Details of studies reporting carriage of *Streptococcus pneumoniae*

| **Reference** | | **Study design** | **Study period** | **Country** | **Setting** | **Sample size** | **Number of swabs** | **Route of swab (Type of swab)** | **Identification method** **(Culture plate)** | **Denominator; Prevalence** | **Age group** | **Prevalence of carriage, % (95% CI)** |
| --- | --- | --- | --- | --- | --- | --- | --- | --- | --- | --- | --- | --- |
| ***Low income countries*** | | | | | | | | | | | | |
| **Healthy population** | | | | | | | | | | | | |
| [65] | Saha et al. 2003 | Cross-sectional | 1999–2000 | Bangladesh | Rural. Outpatient department | 2839 children | 2839 | Nasopharyngeal -anterior nasal  (cotton (Cotton) | Microbiology (sheep blood agar + 5 µg/mL gentamicin 5 µg/mL) | Persons; Point prevalence | 0–1 month | 26 |
|  |  |  |  |  |  |  |  |  |  |  | 2–5 months | 36 |
|  |  |  |  |  |  |  |  |  |  |  | 6–11 months | 42 |
|  |  |  |  |  |  |  |  |  |  |  | 12–23 months | 50 |
|  |  |  |  |  |  |  |  |  |  |  | 24–35 months | 51 |
|  |  |  |  |  |  |  |  |  |  |  | 36–48 months | 46 |
|  |  |  |  |  |  |  |  |  |  |  | ≥48 months | 47 |
|  |  |  |  |  |  |  |  |  |  |  | Total | 46 |
| [49] | Granat et al. 2007 | Prospective community-based longitudinal | 2000–2001 | Bangladesh | Rural. Households | 98 families with 99 new-borns) | 4324 | Nasopharyngeal (Calcium alginate, WHO) ^a^ | Microbiology (sheep blood + 5 µg/mL gentamicin) | Samples; Average prevalence | 4–12 months | 49.3 |
|  |  |  |  |  |  |  |  |  |  |  | 1–4 years | 50.9 |
|  |  |  |  |  |  |  |  |  |  |  | 5–9 years | 41.5 |
|  |  |  |  |  |  |  |  |  |  |  | 10–18 years | 32.4 |
|  |  |  |  |  |  |  |  |  |  |  | Mothers | 7.3 |
|  |  |  |  |  |  |  |  |  |  |  | Other adults | 8.2 |
| [116] | Coles et al. 2011 | Cross-sectional | 2005–2007 | Bangladesh | Rural. 2 villages | 225 children | 225 | Nasopharyngeal (Calcium alginate) | Microbiology (5% sheep blood + 5 µg/mL gentamicin) | Persons; Point prevalence | 12 weeks | 72.9 |
| [42] | Cheung et al. 2009 | Cohort | 2003 | The Gambia | Urban/rural: NR. Government vaccination | 2342 children | 4189 | Nasopharyngeal (WHO) ^a^ | Microbiology (gentamicin blood agar) and confirmation with PCR | Samples; Average prevalence | 9-15 months | 86.2 ^b^ |
|  |  |  |  |  |  |  |  |  |  |  | 21-27 months | 84.6 ^b^ |
|  |  | Cross-sectional | 2003–2004 |  |  | 675 of their younger siblings | 675 |  |  | Samples; Point prevalence | Median age: 3 months | 92.7 ^b^ |
| [53] | Roca et al. 2011 | Double-blind, cluster randomized controlled | 2003–2008 (pre-vaccination: 2003–2004) | The Gambia | Rural.  21 villages | 2094 individuals pre-vaccination | 2094 | Nasopharyngeal (Calcium alginate) | Microbiology (gentamicin blood agar) | Persons; Point prevalence | 2–<5 years | 93.4 ^b^ |
|  |  |  |  |  |  |  |  |  |  |  | 5–<15 years | 86.3 |
|  |  |  |  |  |  |  |  |  |  |  | ≥15 years | 60.6 |
| [43] | Kwambana et al. 2011 | Longitudinal | NR | The Gambia | NR | 30 infants | 498 | Nasopharyngeal (Calcium alginate) | Molecular (*cpsA* PCR) (gentamicin blood agar) | Samples; Average prevalence | 0–12 months | 78 (76–83) |
| [54] | Hill et al. 2010 | Longitudinal | NR | The Gambia | Rural.  2 villages | 158 individuals | 1522 | Nasopharyngeal (WHO) ^a^ | Microbiology  (5 µg/mL gentamicin blood agar) | Persons; Period prevalence | Children | 97 |
|  |  |  |  |  |  |  |  |  |  |  | Adults | 85 |
| [50] | Hill et al. 2008 | Longitudinal | NR | The Gambia | Rural. 21 villages | 236 infants | 3145 | Nasopharyngeal (WHO) ^a^ | Microbiology (5 µg/mL gentamicin sheep blood agar) | Persons; Period prevalence | 0–11 months | 20 |
|  |  |  |  |  |  |  |  |  |  |  | 0.25 months | 90 |
|  |  |  |  |  |  |  |  |  |  |  |  | 100 (9-month period) |
| [57] | Hill et al. 2006 | Cross-sectional | NR | The Gambia | Rural.  21 villages | 2972 individuals | 2872 | Nasopharyngeal (WHO) ^a^ | Microbiology  (5 µg/mL gentamicin sheep blood agar) | Persons; Point prevalence | Median age: 15 years | 72 |
| [39] | Lloyd-Evans et al. 1996 | Case-control (cross-sectional for carriage) | 1989–1991 | The Gambia | Urban/rural: NR. Government health centers | 113 children | 113 | Nasopharyngeal (Cotton) | Microbiology  (5 µg/mL gentamicin sheep blood agar) | Persons; Point prevalence | <5 years | 76.1 |
| [55] | Abdullahi et al. 2008 | Longitudinal | 2004 | Kenya | Semi-urban and rural.  4 villages | 450 individuals | 864 | Nasopharyngeal (Rayon, WHO) ^a^ | Microbiology (7% horse blood agar + 2.5 µg/mL gentamicin and 7% chocolate agar) | Persons; Average prevalence | Total |  |
|  |  |  |  |  |  |  | 98 |  |  |  | <1 years | 59 (49–69) |
|  |  |  |  |  |  |  | 130 |  |  |  | 1–2 years | 61 (52–69) |
|  |  |  |  |  |  |  | 121 |  |  |  | 3–4 years | 50 (41–60) |
|  |  |  |  |  |  |  | 109 |  |  |  | 5–9 years | 41 (32-51) |
|  |  |  |  |  |  |  | 104 |  |  |  | 10–19 years | 9.6 (4.7–17) |
|  |  |  |  |  |  |  | 102 |  |  |  | 20–29 years | 7.8 (3.4–15) |
|  |  |  |  |  |  |  | 93 |  |  |  | 30–49 years | 3.2 (0.7–9.1) |
|  |  |  |  |  |  |  | 107 |  |  |  | ≥50 years | 4.7 (1.5–11) |
|  |  |  |  |  |  |  |  |  |  |  | 0–4 years | 57 |
|  |  |  |  |  |  |  |  |  |  |  | 10–85 years | 6.4 |
| [52] | Abdullahi et al. 2012 | Longitudinal | 2006–2009 | Kenya | Kilifi Health and Demographic Surveillance System (KHDSS) | 2840 children | 9466 | Nasopharyngeal (Dacron, WHO) ^a^ | Microbiology  (Blood agar + 2.5 µg/mL gentamicin) | Persons; Point prevalence | 3–59 months | 65.8 |
| [51] | Tigoi et al. 2012 | Longitudinal | 2006–2009 | Kenya | Urban/rural: NR. Maternity department and immunization clinic | 1404 children 1372 mothers 221 fathers 1412 siblings | 12610 | Nasopharyngeal (Dacron), WHO) ^a^ | Microbiology (Blood agar 2.5 µg/mlmlmLml gentamicin) | Persons; Average prevalence | Mean: 2.1 days Mean age family members: NR | 63.2 ^c^ |
| [64] | Valles et al. 2006 | Cross-sectional | 2003 | Mozambique | Rural. Hospital outpatient department | 285 children | 285 | Nasopharyngeal (Calcium alginate) | Microbiology (Blood agar + 5 µg/mL gentamicin) | Persons; Point prevalence | <5 years | 87 |
| [38] | Coles et al. 2008 | Prospective, matched case-control (nested in a community randomized trial) | 2003–2005 | Nepal | Rural. Households | 197 children ^a^ | 197 | Nasopharyngeal (Rayon) | Microbiology (Blood agar + 5% sheep blood and 2.5 µg/mL gentamicin) | Persons; Point prevalence | 1–36 months | 78.7 |
| [63] | Moyo et al. 2012 | Cross-sectional | 2010 | Tanzania | Urban/rural: NR. Child health clinic | 300 children | 300 | Nasopharyngeal (Rayon) | Microbiology (5% sheep blood agar ± gentamicin) | Persons; Point prevalence | <5 years | 35 |
| **Immunocompromised population** | | | | | | | | | | | | |
| [117] | Rusen et al. 1997 | Cross-sectional | 1990 | Kenya | Urban.  Labor and delivery ward | 26 children with HIV | NR | Nasopharyngeal (Calcium alginate) | Microbiology (Chocolate blood agar) | Persons; Point prevalence | <5 years | 86 |
| [58] | Abdullahi et al. 2012 | Repeated cross-sectional | 2006-2008 | Kenya | Urban/rural: NR.  HIV clinic | 99 children with HIV | 99 | Nasopharyngeal (Dacron) | Microbiology (Blood agar + 2.5 µg/mL gentamicin) | Persons; Point prevalence | 3–59 months | 76 (66-84) |
| [41] | Anthony et al. 2012 | Cross-sectional | 2008 | Tanzania | Rural.  HIV clinic | 142 children with HIV | 142 | Nasopharyngeal (WHO) ^a^ | Microbiology and culture-negative samples tested by *lytA* PCR | Persons; Point prevalence | 1–4 years | 88 |
|  |  |  |  |  |  |  |  |  |  |  | 5–9 years | 77 |
|  |  |  |  |  |  |  |  |  |  |  | 10–14 years | 76 |
| [59] | Kateete et al. 2012 | Cross-sectional | 2001–2002 | Uganda | Urban.  Sickle Cell clinic | 81 children with homozygote sickle cell disease | 81 | Nasopharyngeal (Calcium alginate) | Microbiology (5% rabbit blood agar) | Persons; Point prevalence | 8 months–6 years | 33 |
| [40] | Blossom et al. 2006 | Cross-sectional | 2004–2005 | Uganda | Urban.  HIV clinic | 600 individuals with HIV | 600 | Oropharyngeal (BBL) | Microbiology (Trypticase soy agar + 5% whole sheep blood) | Persons; Point prevalence | Adults (mean age 38.15 years) | 18 |
| **Sick population** | | | | | | | | | | | | |
| [39] | Lloyd-Evans et al. 1996 | Case-control (cross-sectional for carriage) | 1989–1991 | The Gambia | Urban/rural: NR. Government health centers | 1152 children: 1071 sick (clinical diagnosis of pneumonia, meningitis, septicemia, or other serious bacterial infection) | 1152 | Nasopharyngeal (Cotton) | Microbiology (5 µg/mL gentamicin sheep blood agar) | Persons; Point prevalence | <5 years | 85.1 |
|  |  |  |  |  |  | 81 children with IPD |  |  |  |  |  | 90.1 |
| [38] | Coles et al. 2008 | Prospective, matched case-control (nested in a community randomized trial) | 2003–2005 | Nepal | Rural. Households | 197 children with ALRI* | 394 | Nasopharyngeal (Rayon) | Microbiology (5% sheep blood + 2.5 µg/mL gentamicin) | Persons; Point prevalence | 1–36 months | 80.2 |
| ***Lower-middle income countries*** | | | | | | | | | | | | |
| **Healthy population** | | | | | | | | | | | | |
| [68] | Russell et al. 2006 | Cross-sectional | 2003–2004 | Fiji | Urban and rural. 8 and 11 villages | 774 children | 440 | Nasopharyngeal (Cotton) | Microbiology (2.5 µg/mL gentamicin 5% sheep blood Columbia agar) | Samples; Point prevalence | 3–13 months | 44.3 |
| [56] | Regev-Yochay et al. 2012 | Cross-sectional | 2009 | Gaza strip | Urban/rural: NR | 379 children | 379 | Nasopharyngeal (Rayon) | Microbiology (Tryptic soy agar + sheep blood and 5 µg/ml gentamicin) | Persons; Point prevalence | 3 weeks–5.5 years | 50 |
|  |  |  |  |  | 12 neighborhoods/ villages | 379 parents | 376 |  |  |  | NR | 8 |
| [66] | Denno et al. 2002 | Cross-sectional | 1996 | Ghana | Urban/rural: NR. Polyclinic and immunization clinic | 311 children | 311 | Nasopharyngeal (Culturette) | Microbiology  (sheep blood agar) | Persons; Point prevalence | 6–12 months | 51.4 |
| [118] | Donkor et al. 2010 | Cross-sectional | 2006–2007 | Ghana | Urban/rural: NR.  Child health department | 124 children | 124 | Nasopharyngeal (NR) | Microbiology (Blood agar + 5% sheep blood agar) | Persons; Point prevalence | <13 years | 15.3 |
| [119] | Coles et al. 2001 | Randomized, double-blind, placebo-controlled | 1998–1999 | India | Rural.  2 villages | 225 infants (placebo arm) | 225 | Nasopharyngeal (Rayon) | Microbiology  (Tryptic soy agar + 5% sheep blood and 5 µg/mL gentamycin) | Samples; Point prevalence | 2 months | 54.2 |
|  |  |  |  |  |  |  | 197 |  |  |  | 4 months | 67.9 |
|  |  |  |  |  |  |  | 171 |  |  |  | 6 months | 69.8 |
| [75] | Das et al. 2002 | Cross-sectional | 2000–2001 | India | Urban.  Schools | 566 children | 566 | Nasopharyngeal (NR) | Microbiology (Modified chocolate agar + 5% sheep blood agar) | Persons; Point prevalence | 5–12 years | 29.1 |
| [120] | Devi et al. 2012 | Cross-sectional | 2009–2010 | India | Rural.  30 villages | 811 children | 811 | Nasopharyngeal (NR) | Microbiology (Blood agar + 5% sheep blood agar) | Persons; Point prevalence | 0–14 years | 12.8 |
| [67] | Rupa et al. 2012 | Longitudinal | 2009–2010 | India | Rural.  90 villages | 210 children | 1679 | Nasopharyngeal (Calcium alginate) | Microbiology (Trypticase soy base + 5% sheep blood agar ± 5 μg/mL gentamicin) | Persons; Point prevalence | 0–1 years | Maximum: 46.3 |
| [121] | Wattal et al. 2007 | Cross-sectional | NR | India | Urban. Pediatric outpatient department | 200 children | 200 | Nasopharyngeal (Calcium alginate) | Microbiology (Trypticase soy agar + 5% sheep blood and 5 µg/mL gentamicin) | Persons; Point prevalence | 3 months–3 years | 6.5 |
| [69] | Soewignjo et al. 2001 | Cross-sectional | 1997 | Indonesia | Rural.  20 randomly selected settlements | 484 children | 484 | Nasopharyngeal (Calcium alginate) | Microbiology (Trypticase soy agar + 5% sheep blood) | Persons; Point prevalence | 0–25 months | 48 (42–54) ^d^ |
| [122] | Adetifa et al. 2012 | Cross-sectional | NR | Nigeria | Rural.  Health center | 1005 individuals | 1005 | Nasopharyngeal (Calcium alginate, WHO) ^a^ | Microbiology (gentamicin 5% sheep blood agar) | Persons; Point prevalence | All ages Median: 4.4 years | 52.5 (49.4–55.7) |
| [36] | Vu et al. 2011 | Case-control | 2007–2008 | Vietnam | Urban/rural: NR. Pediatric department | 350 children | NR | Nasopharyngeal (Dacron, WHO) ^a^ | Molecular (PCR) | Persons; Point prevalence | <5 years | 50.3 |
| [123] | Gill et al. 2008 | Two-arm longitudinal | 2003–2005 | Zambia | Urban. Antenatal clinics | 132 children born to HIV positive mothers and 128 children born to HIV negative mothers | 1394 | Nasopharyngeal (Calcium alginate) | Microbiology  (Soy trypticase agar + 5% sheep blood and 5% gentamicin) | Samples; Average prevalence | 6 weeks | 25.8 |
| **Immunocompromised population** | | | | | | | | | | | | |
| [70] | Bhattacharya et al. 2012 | Cross-sectional | 2008–2009 | India | Urban/rural: NR. Outpatient care at pediatric HIV clinic | 148 children with HIV | 148 | Nasopharyngeal (Calcium alginate) | Microbiology  (Sheep blood agar + 5 µg/mL gentamicin and chocolate agar + 300 µg/mL bacitracin) | Persons; Point prevalence | 1–16 years | 28 |
| [71] | Mwenya et al. 2010 | Double-blind, randomized controlled | 2002–2003 | Zambia | Urban. Suburbs | 439 children with HIV | 630 | Nasopharyngeal (Rayon) | Microbiology (5% blood agar + 5 µg/mL gentamicin) | Persons; Point prevalence | 6 months–14 years | 51 |
| [124] | Gill et al. 2008 | Longitudinal cohort | 2003–2005 | Zambia | Urban/rural: NR. Antenatal clinics. | 132 women with HIV | 1402 | Nasopharyngeal (Calcium alginate) | Microbiology (Soy trypticase agar + 5% sheep blood and 5% gentamicin) | Samples; Average prevalence | Mean: 25.9 years | 11.4 |
| **Sick population** | | | | | | | | | | | | |
| [62] | Mastro et al. 1993 | Cross-sectional | 1989–1990 | Pakistan | Rural. Outpatient clinics, ER and immunization clinics. | 601 children with ARI | 601 | Nasopharyngeal (Dacron) | Microbiology (Trypticase soy agar + 5% sheep blood and 5 µg/mL gentamicin and chocolate agar + 300 µg/mL bacitracin) | Persons; Point prevalence | Mean: 14.5 months | 64.4 |
| [60] | Lankinen et al. 1994 | Cross-sectional | 1984 | The Philippines | Urban Hospital | 318 children with ALRI | 227 | Nasopharyngeal aspirates | Microbiology (3x blood agar: without antibiotics, + gentamicin, or + bacitracin and MacConkey agar) | Samples; Point prevalence | <5 years | 51 |
| [61] | Lupisan et al. 2000 | Cross-sectional | 1994 | The Philippines | Rural | 956 children with severe pneumonia, suspected meningitis or clinical suspicion of sepsis. | 935 | Nasopharyngeal (Cotton) | Microbiology (Sheep blood agar + 5 µg/mL gentamicin and chocolate agar + 100 µg/mL bacitracin) | NR; Point prevalence | 0–59 months | 27.9 |
|  |  |  |  |  | Tertiary care government hospital. |  |  |  |  |  |  |  |
| [36] | Vu et al. 2011 | Case-control | 2007–2008 | Vietnam | Urban/rural: NR. Pediatric department | 274 children with radiologically confirmed pneumonia | NR | Nasopharyngeal (Dacron, WHO) ^a^ | Molecular (PCR) | Persons; Point prevalence | <5 years | 38.7 |
|  |  |  |  |  |  | 276 children with other LRTI |  |  |  |  |  | 43.3 |

HIV, human immunodeficiency virus; LRTI, lower respiratory tract infection; NR, not reported; WHO, World Health Organization.

^a^ Sampling, storage, and culture procedures were done according to the WHO guidelines.[44,45]

^b^ All carriage rates presented are from the control group.

^c^ Overall carriage rates. Children, mothers, fathers, and siblings combined.

^d^ Age- and population-weighted carriage rate, adjusted for design effect.
