# Supplementary material for: Carriage of Streptococcus pneumoniae and Other Respiratory Bacterial Pathogens in Low and Lower-Middle Income Countries: A Systematic Review and Meta-Analysis
Source: PLoS One. 2014 Aug 1;9(8):e103293. doi: 10.1371/journal.pone.0103293 (PMC4118866; doi:10.1371/journal.pone.0103293)
Supplement: Table S3 — Studies reporting carriage of individual pneumococcal serotypes or serogroups. (DOCX) [file pone.0103293.s003.docx]

**Table S3:** Studies reporting carriage of individual pneumococcal serotypes or serogroups

| **Reference** | | **Study period** | | **Country** | **Study population** | **Serogroup/ serotype** | **Denominator** | **Five most frequent serotypes/serogroups** |
| --- | --- | --- | --- | --- | --- | --- | --- | --- |
| ***Low income countries*** | | | | | | | | |
| **Healthy population** | | | | | | | | |
| [49] | Granat et al. 2007 | 2000-2001 | Bangladesh | | 99 newborns (age: NR) | Serotypes | Isolates | 19F: 11.5% 6A: 10.1% 6B: 8.0% 23F: 4.9% 19A: 3.7% |
| [42] | Cheung et al. 2009 | 2003-2004 | The Gambia | | Children (median age: 12 months) | Serotypes | Persons | 6A: 15.8% ^a^ 19F: 13.8% 6B: 8.5% 15: 7.8% 23F: 7.4% |
| [53] | Roca et al. 2011 | 2003-2008 | The Gambia | | Individuals (age: 2–54 years) | Serotypes | Persons | 3: 8.7% ^b^ 23F: 4.1% 6A: 4.0% 11: 3.9% 6B: 3.8% |
| [46] | Akinsola et al. 2012 | 2005-NR | The Gambia | | Children (mean age at 1^st^ visit: 45 months) | Serotypes | Persons | 23F: 5% ^c^ 6B: 5% 14: 3% 19F: 2% 4: 1.4% |
| [50] | Hill et al. 2008 | NR | The Gambia | | Children (age: NR) | Serotypes | Isolates | 6B:14.4% 19F:10.4% 6A: 8.9% 14: 8.6% 23F: 8.3% |
| [57] | Hill et al. 2006 | NR | The Gambia | | Individuals (mean age: 15 years) | Serotypes | Isolates | 3: 9% 6A: 6%  23F: 6% 6B: 5% 19F: 5% |
| [47] | Ota et al. 2011 | NR | The Gambia | | Infants (age: 6–8 weeks) | Serogroups | Persons | PCV-7: 10.0%–20.2% Non-PCV-7: 53.0%-72.7% |
| [55] | Abdullahi et al. 2008 | 2004 | Kenya | | Individuals (age: 0–85 years) | Serotypes | Isolates | 19F: 14% 6A: 8.6% 23F: 8.2% 6B: 7.9% 23B: 5.4% |
| [58] | Abdullahi et al. 2012 | 2006-2008 | Kenya | | Children (age 3–59 months) | Serotypes | Persons | 19F: 10% 6A: 8.3% 6B: 6.5% 23F: 4.1% 11A: 3.2% |
| [51] | Tigoi et al. 2012 | 2006-2009 | Kenya | | Infants (age: 0–13 weeks) | Serotypes | Isolates | 19F: 13.4% 6A: 7.9% 6B: 6.7% 23F: 6.5% 23B: 5.3% |
| [64] | Valles et al. 2006 | 2003 | Mozambique | | Healthy children (age<5 years) attending outpatient department | Serotypes | Isolates | 19F: 26%  19A: 8% 23F: 7% 6A: 7% 6B: 6% |
| [63] | Moyo et al. 2012 | 2010 | Tanzania | | Healthy children (age <5 years) | Serotypes | Isolates | 19F: 21.7% 6B 13.0% 9V: 12.2% 13: 12.2% |
| **Immunocompromised population** | | | | | | | | |
| [58] | Abdullahi et al. 2012 | 2006-2008 | | Kenya | HIV-infected children (age: 3–59 months) | Serotypes | Persons | 19F: 14% 6A: 13% 23F: 9% 6B: 5% 3: 4% |
| [41] | Anthony et al. 2008 | 2008 | | Tanzania | HIV-positive children (age: 1–14 years) | Serotypes | Isolates | 19F: 11% 11A: 9% 6B: 6.1% 23F: 6.1% 14: 6.1% 3: 6.1% |
| [40] | Blossom et al. 2006 | 2004-2005 | | Uganda | HIV infected adults (median age: 38 years) | Serotypes | Isolates | 3: 14.7% 19F: 6.4% 23F: 6.4% 16: 5.5% |
| **Sick population** | | | | | | | | |
| [39] | Lloyd-Evans et al. 1996 | 1989-1991 | | The Gambia | Children with clinical diagnosis of pneumonia, septicemia, meningitis, or other serious infections (age: <5 years) | Serogroups | Persons | 6: 21.3% 19: 18.5% 14: 11.9% 9: 7.1% 23: 7.0% |
| ***Lower-middle income countries*** | | | | | | | | |
| **Healthy population** | | | | | | | | |
| [68] | Russel et al. 2006 | 2003-2004 | | Fiji | Children (age 3–13 months) | Serotypes | Isolates | 6A:13.0% 23F: 8.4% 19F: 7.5% 6B: 6.3% 15B: 5.0% |
| [69] | Soewignjo et al. 2001 | 1997 | | Indonesia | Infants (age 0–25 months) | Serogroups | Isolates | 6: 25% 23: 21% 33: 8.1% 15: 7.6% 19: 5.9% |
| [122] | Adetifa et al. 2012 | NR | | Nigeria | Individuals (median age: 4.4 years) | Serotypes | Isolates | 19F: 17.8% 6A: 14.4% 6B: 13.6% 23F: 7.0% 11: 5.1% |
| [123] | Gill et al. | 2003-2005 | | Zambia | Infants born to HIV-positive women and control infants born to HIV-negative women (age: NR) | Serotypes | Isolates | 19F:16.0% 6B: 9.9% 23F: 7.5% 15: 7.0%  14: 6.4% |
| [62] | Mastro et al. 1993 | 1989-1990 | | Pakistan | Healthy urban children (mean age: 11.0, months) | Serotypes | Isolates | 19F: 14.3% 25F: 14.3% 19A: 8.2% 6A: 6.1% 5: 6.1% |
|  |  |  | |  | Rural children (mean age: 21.7 months) | Serotypes | Isolates | 19F: 14.4% 10: 14.4% 6A: 7.6% 6B: 7.6% 34: 6.8% |
| **Immunocompromised population** | | | | | | | | |
| No data found. | | | | | | | | |
| **Sick population** | | | | | | | | |
| [62] | Mastro et al. 1993 | 1989-1990 | | Pakistan | Urban children with ARI (mean age: 14.5 months) | Serotypes | Isolates | 19B: 12.3% 6A: 9.8% 19A: 7.6% 18C: 7.3% 23F: 6.5% |
| [60] | Lankinen et al. 1994 | 1984 | | The Philippines | Children with ALRI (age <5 years) | Serogroups | Isolates | 6: 17.4% 19: 14.7% 14: 14.7% 15: 3.8% 23: 8.7% |
| [48] | Bogaert et al. | 1997-1999 | | Vietnam | Children with URTI (age<5 years) | Serotypes | Isolates | 23F: 32% 19F: 21% 6B: 13%  14: 10% 6A: 4% 15: 4% |
| [36] | Vu et al. 2011 | 2007-2008 | | Vietnam | Children with RCP or other LRTI, and controls (age<5 years) | Serotypes | Isolates | 6A/B, 19F, 23F, 14, 15B/C 19F-RCP: 38.7% 19F-LRTI : 23.5% 19F-controls: 15.9% |

ALRI, acute lower respiratory infection; ARI, acute respiratory infection; HIV, human immunodeficiency virus; IPD, invasive pneumococcal disease; LRTI, lower respiratory tract infection; NR, not reported; PCV-9, 9-valent pneumococcal conjugate vaccine; RCP, radiologically confirmed pneumonia; URTI, lower respiratory tract infection.

^a^ Data are from the control group (vaccinated with placebo) at 9 to 15 months of age.

^b^ Data are pooled from both groups before vaccination with PCV-9.

^c^ Data are from the control children (vaccinated with placebo) before vaccination with PCV-7
