# Supplementary material for: Carriage of Streptococcus pneumoniae and Other Respiratory Bacterial Pathogens in Low and Lower-Middle Income Countries: A Systematic Review and Meta-Analysis
Source: PLoS One. 2014 Aug 1;9(8):e103293. doi: 10.1371/journal.pone.0103293 (PMC4118866; doi:10.1371/journal.pone.0103293)
Supplement: Table S4 — Details of studies reporting carriage of Haemophilus influenzae . (DOCX) [file pone.0103293.s004.docx]

**Table S4.** Details of studies reporting carriage of *Haemophilus influenzae*

| **Reference** | | **Study design** | **Study period** | **Country** | **Setting** | **Sample size** | **Number of swabs** | **Route of swab (Type of swab)** | **Identification method (Culture plate)** | **Denominator; Prevalence** | **Age group** | **Prevalence of carriage, % (95% CI)** |
| --- | --- | --- | --- | --- | --- | --- | --- | --- | --- | --- | --- | --- |
| ***Low income countries*** | | | | | | | | | | | | |
| **Healthy population** | | | | | | | | | | | | |
| [43] | Kwambana et al. 2011 | Longitudinal | NR | The Gambia | Rural. 21 villages. | 30 infants | 498 | Nasopharyngeal (Calcium alginate) | Molecular (*OmpP2* PCR) | Samples; Average prevalence | 0–12 months | 70 (65–74) |
| [72] | Adegbola et al. 1998 | Double-blind, randomized, placebo controlled | NR | The Gambia | Urban and rural. Immunization centers | 3986 children | 3986 | Oropharyngeal (Cotton) | Microbiology (Levinthal agar ASA + burro anti-Hib antiserum, 5 U/mL bacitracin, 3.9 µg/mL vancomycin, 0.78 µg/mL clindamycin) | Persons; Point prevalence | 1–2 years | Urban: 5.6 ^a^ Rural: 10.9 |
| [55] | Abdullahi et al. 2008 | Longitudinal | 2004 ^b^ | Kenya | Semi-urban and rural.  4 villages | 450 individuals | 864 | Nasopharyngeal (Rayon) | Microbiology (7% horse blood agar + 2.5 µg/mL gentamicin and 7% chocolate agar) | Persons; Point prevalence | Total |  |
|  |  |  |  |  |  |  | 98 |  |  |  | <1 years | 29 (20–39) |
|  |  |  |  |  |  |  | 130 |  |  |  | 1–2 years | 28 (20–36) |
|  |  |  |  |  |  |  | 121 |  |  |  | 3–4 years | 21 (15–30) |
|  |  |  |  |  |  |  | 109 |  |  |  | 5–9 years | 24.0 (16–33) |
|  |  |  |  |  |  |  | 104 |  |  |  | 10–19 years | 3.8 (1.1–9.6) |
|  |  |  |  |  |  |  | 102 |  |  |  | 20–29 years | 3.9 (1.1–9.7) |
|  |  |  |  |  |  |  | 93 |  |  |  | 30–49 years | 1.1 (0.03–5.8) |
|  |  |  |  |  |  |  | 107 |  |  |  | ≥50 years | 2.8 (0.6–8.0) |
|  |  |  |  |  |  |  |  |  |  |  | 0–4 years | 26.0 |
|  |  |  |  |  |  |  |  |  |  |  | 10–85 years | 3.0 |
| [74] | Williams et al. 2011 | Cross-sectional | 2007 | Nepal | Urban. Outpatient department, schools and children’s homes | 2195 children: | 2195 | Oropharyngeal (Cotton) | Microbiology (Hib antiserum agar) | Persons; Point prevalence | 0–13 years | 5.0 (3.9–6.4) |
|  |  |  |  |  |  |  |  |  |  |  | 3–11 months | 3.4 (2.5–4.7 |
|  |  |  |  |  |  |  |  |  |  |  | 1–4 years | 6.4 (4.7–8.8) |
|  |  |  |  |  |  |  |  |  |  |  | ≥5 years | 5.0 (3.1–8.1) |
|  |  |  |  |  |  | 1311 outpatient department |  |  |  |  | 0–12 years | 4.1 |
|  |  |  |  |  |  | 647 schools |  |  |  |  | 3–13 years | 5.4 |
|  |  |  |  |  |  | 237 children’s homes |  |  |  |  | 0–12 years | 8.9 |
| **Immunocompromised population** | | | | | | | | | | | | |
| No data found | | | | | | | | | | | | |
| **Sick population** | | | | | | | | | | | | |
| No data found | | | | | | | | | | | | |
| ***Lower-middle income countries*** | | | | | | | | | | | | |
| **Healthy population** | | | | | | | | | | | | |
| [75] | Das et al. 2002 | Cross-sectional | 2000–2001 | India | Urban.  Schools | 566 children | 566 | Nasopharyngeal (NR) | Microbiology (Modified chocolate agar and 5% sheep blood agar) | Persons; Point prevalence | 5–12 years | 28.6 |
| [76] | Sekhar et al. 2009 | Cross-sectional | 2005–2006 | India | Rural and urban.  Random households | 1000 children | 1000 | Nasopharyngeal (Calcium alginate) | Microbiology (Chocolate agar + 300 μg/mL bacitracin) | Persons; Point prevalence | <2 years | 11.2 |
| [73] | Gessner et al. 1998 | Cross-sectional | NR | Indonesia | Rural.  20 randomly selected settlements | 484 children | 484 | Nasopharyngeal (WHO) | Microbiology (Chocolate agar + 300 µg/mL bacitracin) | Persons; Point prevalence | 0–24 months | 4.6 (3.7–5.5) ^c^ |
| [36] | Vu et al. 2011 | Case-control | 2007–2008 | Vietnam | Urban/rural: NR. Pediatric department | 350 children | NR | Nasopharyngeal (Dacron, WHO) | Molecular (PCR) | Persons; Point prevalence | <5 years | 31.4 |
| **Immunocompromised population** | | | | | | | | | | | | |
| [70] | Bhattacharya et al. 2012 | Cross-sectional | 2008–2009 | India | Urban/rural: NR.  Outpatient care at pediatric HIV clinic | 148 children with HIV | 148 | Nasopharyngeal (Calcium alginate) | Microbiology (Sheep blood agar + 5 µg/mL gentamicin and chocolate agar + 300 µg/mL bacitracin) | Persons; Point prevalence | 1–16 years | 24 |
| [71] | Mwenya et al. 2010 | Double-blind, randomized control study | 2002–2003 | Zambia | Urban. Suburbs | 439 children with HIV | 630 | Nasopharyngeal (Rayon) | Microbiology (chocolate agar + 5 µg/mL vancomycin) | Persons; Point prevalence | 6 months–14 years | 29 |
| **Sick population** | | | | | | | | | | | | |
| [62] | Mastro et al. 1993 | Cross-sectional | 1989–1990 | Pakistan | Rural. Outpatient clinics, emergency departments and immunization clinics | 601 children with ARI | 601 | Nasopharyngeal (Dacron) | Microbiology (Trypticase soy agar + 5% sheep blood, 5 µg/mL gentamicin and chocolate agar + 300 µg/mL bacitracin) | Persons; Point prevalence | Mean: 14.5 months | 36.6 |
| [61] | Lupisan et al. 2000 | Cross-sectional | 1994 | The Philippines | Rural.  Tertiary care government hospital | 956 children with severe pneumonia, suspected meningitis, or clinical suspicion of sepsis. | 935 | Nasopharyngeal (Cotton) | Microbiology (Sheep blood agar + 5 µg/mL gentamycin and chocolate agar + 100 µg/mL bacitracin) | NR; Point prevalence | 0–59 months | 15.2 |
| [36] | Vu et al. 2011 | Case-control | 2007–2008 | Vietnam | Urban/rural: NR. Pediatric department | 274 children with radiologically confirmed pneumonia | NR | Nasopharyngeal (Dacron, WHO) | Molecular (PCR) | Persons; Point prevalence | <5 years | 50 |
|  |  |  |  |  |  | 276 children with other LRTI |  |  |  |  |  | 53.5 |

ARI, acute respiratory infection; HIV, human immunodeficiency virus; LRTI, lower respiratory tract infection; PCR, polymerase chain reaction; NR, not reported.

^a^ All carriage rates presented are from the control group.

^b^ Post-Hib vaccination data

^c^ Age- and population-weighted carriage rate, adjusted for design effect.
