# Supplementary material for: Carriage of Streptococcus pneumoniae and Other Respiratory Bacterial Pathogens in Low and Lower-Middle Income Countries: A Systematic Review and Meta-Analysis
Source: PLoS One. 2014 Aug 1;9(8):e103293. doi: 10.1371/journal.pone.0103293 (PMC4118866; doi:10.1371/journal.pone.0103293)
Supplement: Table S5 — Details of studies reporting carriage of Moraxella catarrhalis . (DOCX) [file pone.0103293.s005.docx]

**Table S5.** Details of studies reporting carriage of *Moraxella catarrhalis*

| **Reference** | | **Study design** | **Study period** | **Country** | **Setting** | **Sample size** | **Number of swabs** | **Route of swab (Type of swab)** | **Identification method (Culture plate)** | **Denominator; Prevalence** | **Age group** | **Prevalence of carriage, % (95% CI)** |
| --- | --- | --- | --- | --- | --- | --- | --- | --- | --- | --- | --- | --- |
| ***Low income countries*** | | | | | | | | | | | | |
| **Healthy population** | | | | | | | | | | | | |
| [43] | Kwambana et al. 2011 | Longitudinal | NR | The Gambia | Urban/rural: NR. 21 villages | 30 infants | 498 | Nasopharyngeal (NR) | Molecular (*copB* PCR) | Samples; Average prevalence | 0–12 months | 71 (67–75) |
| **Immunocompromised population** | | | | | | | | | | | | |
| No data found | | | | | | | | | | | | |
| **Sick population** | | | | | | | | | | | | |
| No data found | | | | | | | | | | | | |
| ***Lower-middle income countries*** | | | | | | | | | | | | |
| **Healthy population** | | | | | | | | | | | | |
| [36] | Vu et al. 2011 | Case-control | 2007–2008 | Vietnam | Urban/rural: NR. Pediatric department | 350 children | NR | Nasopharyngeal (Dacron, WHO) | Molecular (PCR) | Persons; Point prevalence | <5 years | 58 |
| [78] | Sehgal et al. 1994 | Cross-sectional | 1992–1993 | Yemen | Urban/rural: NR.  Clinic for healthy babies, school, university | 35 children | NR | Nasopharyngeal and oropharyngeal (NR) | Microbiology (Trypticase soy agar + 5-7% human blood, 10 mg/L vancomycin, 5 mg/L trimethoprim, 2 mg/L amphotericin B, and 10 mg/L acetazolamide) | Persons; Point prevalence | <3 years | 31.4 |
|  |  |  |  |  |  | 96 school children |  |  |  |  | 4–12 years | 38.5 |
|  |  |  |  |  |  | 120 university students and staff |  |  |  |  | 20–40 years | 11.7 |
| **Immunocompromised population** | | | | | | | | | | | | |
| No data found | | | | | | | | | | | | |
| **Sick population** | | | | | | | | | | | | |
| [36] | Vu et al. 2011 | Case-control | 2007–2008 | Vietnam | Urban/rural: NR. Pediatric department | 274 children with radiologically confirmed pneumonia | NR | Nasopharyngeal (Dacron, WHO) | Molecular (PCR) (NR) | Persons; Point prevalence | <5 years | 28.1 |
|  |  |  |  |  |  | 276 children with other LRTI |  |  |  |  |  | 42.2 |
| [78] | Sehgal et al. 1994 | Cross-sectional | 1992–1993 | Yemen | Urban/rural: NR. Hospital | 64 children with respiratory infections: | NR | Nasopharyngeal and oropharyngeal (NR) | Microbiology (NR) | Persons; Point prevalence | 3–12 years | 21.9 |
|  |  |  |  |  |  | OM |  |  |  |  |  | 26.9 |
|  |  |  |  |  |  | Sinusitis |  |  |  |  |  | 9.1 |
|  |  |  |  |  |  | Tonsillitis/ Pharyngitis |  |  |  |  |  | 22.2 |

LRTI, lower respiratory tract infection; NR, not reported; PCR, polymerase chain reaction; OM, otitis media.
