# Supplementary material for: Carriage of Streptococcus pneumoniae and Other Respiratory Bacterial Pathogens in Low and Lower-Middle Income Countries: A Systematic Review and Meta-Analysis
Source: PLoS One. 2014 Aug 1;9(8):e103293. doi: 10.1371/journal.pone.0103293 (PMC4118866; doi:10.1371/journal.pone.0103293)
Supplement: Table S6 — Details of studies reporting carriage of Staphylococcus aureus . (DOCX) [file pone.0103293.s006.docx]

**Table S6.** Details of studies reporting carriage of *Staphylococcus aureus*

| **Reference** | | **Study design** | | **Study period** | **Country** | | | **Setting** | | **Sample size** | | **Number of swabs** | | **Route of swab (Type of swab)** | **Identification method (Culture plate)** | **Denominator; Prevalence** | | **Age group** | | **Prevalence of carriage, % (95% CI)** | |
| --- | --- | --- | --- | --- | --- | --- | --- | --- | --- | --- | --- | --- | --- | --- | --- | --- | --- | --- | --- | --- | --- |
| ***Low income countries*** | | | | | | | | | | | | | | | | | | | | | |
| **Healthy population** | | | | | | | | | | | | | | | | | | | | | |
| [43] | Kwambana et al. 2011 | | Longitudinal | NR | | The Gambia | | | Rural. 21 villages | | 30 infants | | 498 | Nasopharyngeal (NR) | Molecular (*nuc* PCR) | | Samples; Average prevalence | | 0–12 months | | 20 (16-24) |
| **Immunocompromised population** | | | | | | | | | | | | | | | | | | | | | |
| [79] | Amir et al. 1995 | | Cross-sectional | 1992 | | Kenya | | | Urban. Community clinic and hospital | | 264 adults with HIV | | 264 | Nasopharyngeal (NR) | Microbiology (5% horse blood agar) | | Persons; Point prevalence | | NR | | 27 |
| **Sick population** | | | | | | | | | | | | | | | | | | | | | |
| No data found | | | | | | | | | | | | | | | | | | | | | |
| ***Lower-middle income countries*** | | | | | | | | | | | | | | | | | | | | | |
| **Healthy population** | | | | | | | | | | | | | | | | | | | | | |
| [80] | Anwar et al. 2004 | | Cross-sectional | 2002–2003 | | | Pakistan | | Urban Private medical center | | 1660 individuals | | 1660 | Nasopharyngeal (Cotton) | Microbiology (Blood and mannitol salt agar) | | Persons; Point prevalence | | All ages | | 14.8 |
|  |  |  |  |  |  |  |  |  |  |  |  |  | 84 |  |  |  |  |  | ≤9 years | | 20.2 |
|  |  |  |  |  |  |  |  |  |  |  |  |  | 209 |  |  |  |  |  | 10–19 years | | 15.8 |
|  |  |  |  |  |  |  |  |  |  |  |  |  | 335 |  |  |  |  |  | 20–29 years | | 13.1 |
|  |  |  |  |  |  |  |  |  |  |  |  |  | 405 |  |  |  |  |  | 30–39 years | | 13.1 |
|  |  |  |  |  |  |  |  |  |  |  |  |  | 353 |  |  |  |  |  | 40–49 years | | 15.6 |
|  |  |  |  |  |  |  |  |  |  |  |  |  | 274 |  |  |  |  |  | ≥50 years | | 16.1 |
| **Immunocompromised population** | | | | | | | | | | | | | | | | | | | | | |
| [70] | Bhattacharya et al. 2012 | | Cross-sectional | 2008–2009 | | | India | | Urban/rural: NR Outpatient care at pediatric HIV clinic | | 148 children with HIV | | 148 | Nasopharyngeal (Calcium alginate) | Microbiology (Sheep blood agar + 5 µg/mL gentamicin and chocolate agar + 300 µg/mL bacitracin) | | Persons; Point prevalence | | 1–16 years | | 26 |
| **Sick population** | | | | | | | | | | | | | | | | | | | | | |
| No data found | | | | | | | | | | | | | | | | | | | | | |

HIV, human immunodeficiency virus; NR, not reported.
