# Supplementary material for: Carriage of Streptococcus pneumoniae and Other Respiratory Bacterial Pathogens in Low and Lower-Middle Income Countries: A Systematic Review and Meta-Analysis
Source: PLoS One. 2014 Aug 1;9(8):e103293. doi: 10.1371/journal.pone.0103293 (PMC4118866; doi:10.1371/journal.pone.0103293)
Supplement: Table S7 — Details of studies reporting carriage of Neisseria meningitidis . (DOCX) [file pone.0103293.s007.docx]

**Table S7.** Details of studies reporting carriage of *Neisseria meningitidis*

| **Reference** | | **Study design** | | **Study period** | **Country** | **Setting** | **Sample size** | **Number of swabs** | **Route of swab (Type of swab)** | **Identification method** **(Culture plate)** | **Denominator; Prevalence** | **Age group** | **Prevalence of carriage, % (95% CI)** |
| --- | --- | --- | --- | --- | --- | --- | --- | --- | --- | --- | --- | --- | --- |
| ***Low income countries*** | | | | | | | | | | | | | |
| **Healthy population** | | | | | | | | | | | | | |
| [84] | Mueller et al. 2007 | | Longitudinal | 2003 | Burkina Faso | Urban.  Clinics | 488 individuals | 2440 | Oropharyngeal (NR) | Molecular (*siaD*, *orf-2* PCR)  (Selective plate) | Persons; Period prevalence | 4–14 years | 18 |
| [85] | Mueller et al. 2008 | | Longitudinal | 2003 | Burkina Faso | Urban households and rural health canters | 488 individuals (urban) | 2327 | Oropharyngeal (NR) | Microbiology (NR) | Samples; Point prevalence | 4–29 years | 6.5 |
|  |  |  | Cross-sectional | 2006 |  |  | 624 individuals (rural) | 617 |  |  |  | 1–39 years | 22 |
| [82] | Kristiansen et al. 2013 | | Cross-sectional (repeated) | 2009–2011 | Burkina Faso | Rural and Urban. Randomly selected households | 20326 individuals | 20326 | Oropharyngeal (NR) | Microbiology (Modified Thayer-Martin VCNT agar + 3 mg/L vancomycin, 7.5 mg/L colistin, 12.5 U/L nystatin, 5 mg/L trimethoprim lactate, and Vitox supplement. | Samples; Point prevalence | 1–29 years | 3.98 |
| [83] | Kristiansen et al. 2011 | | Cross-sectional (repeated) | NR | Burkina Faso | Rural and Urban. Randomly selected households | 20326 individuals | 20326 (5024–5121 per survey) | Oropharyngeal (NR) | Microbiology (Modified Thayer-Martin VCNT agar) | Samples; Point prevalence | 1–29 years | Survey 1: 4.10 Survey 2: 5.27 Survey 3: 3.37 Survey 4: 3.17 |
| [86] | Nicolas et al. 2007 | | Cross-sectional (repeated) | 2003 ^a^ | Niger | Urban/rural: NR. Primary schools | 287 children | NR | Nasopharyngeal (NR) | Microbiology (Chocolate agar + polyvitex, vancomycin, colistin, and nyastatin) | Samples; Point prevalence | NR | Swab 1: 13.2 Swab 2: 10.1 Swab 3: 11.4 |
| [81] | Cheesbrough et al. 1995 | | Cross-sectional | 1990–1991 | Democratic Republic of Congo (formerly Zaire) | Rural.  Hospital and boarding school | 134 children | 134 | Oropharyngeal (NR) | Microbiology (Selective Kellogg’s media) | Samples; Point prevalence | 3–24 months | 0.78 (0-1.7) ^b^ |
|  |  |  |  |  |  |  | 249 children attending boarding school | 249 | Nasopharyngeal (NR) |  |  | 10–14 years | 0.8 (0-1.9) |
| **Immunocompromised population** | | | | | | | | | | | | | |
| No data found | | | | | | | | | | | | | |
| **Sick population** | | | | | | | | | | | | | |
| No data found | | | | | | | | | | | | | |
| ***Lower-middle income countries*** | | | | | | | | | | | | | |
| **Healthy population** | | | | | | | | | | | | | |
| [88] | Leimkugel et al. 2007 | | Cross-sectional (repeated) | 1998-2005 ^a^ | Ghana | Mainly rural.  37 compounds. | 292-350 individuals per survey (16 surveys in total) | 4999 | Pharyngeal  (NR) | Microbiology  (Thayer-Martin agar) | Samples; Point prevalence | All ages | 0.6-19.8 |
| [87] | Ichhpujani et al. 1990 | | Cross-sectional (repeated) | 1986-1987 | India | Urban.  Schools. | 6513 children | 6513 | Nasopharyngeal (West) | Microbiology (Thayer-Martin agar) | Persons; Point prevalence | 6-20 years | 1.64 |
| [89] | Odugbemi et al. 1992 | | Cross-sectional | 1986-1987 | Nigeria | Urban/rural: NR. Primary school. | 639 children | 639 | Nasopharyngeal (Cotton) | Microbiology  (5% heated sheep or human blood [chocolate agar] and modified Thayer-Martin agar) | Persons; Point prevalence | 5-15 years | 6.2 |
| **Immunocompromised population** | | | | | | | | | | | | | |
| No data found | | | | | | | | | | | | | |
| **Sick population** | | | | | | | | | | | | | |
| No data found | | | | | | | | | | | | | |

NR, not reported; PCR, polymerase chain reaction

^a^ Post-meningococcal vaccination data

^b^ All carriage rates presented are from the control group.
