# Supplementary material for: Carriage of Streptococcus pneumoniae and Other Respiratory Bacterial Pathogens in Low and Lower-Middle Income Countries: A Systematic Review and Meta-Analysis
Source: PLoS One. 2014 Aug 1;9(8):e103293. doi: 10.1371/journal.pone.0103293 (PMC4118866; doi:10.1371/journal.pone.0103293)
Supplement: Table S8 — Impact of vaccination on Streptococcus pneumoniae carriage by age. (DOCX) [file pone.0103293.s008.docx]

**Table S8.** Impact of vaccination on *Streptococcus pneumoniae* carriage by age

| **Reference** | | **Country** | **Interventions** | **Group (time or treatment)** | **Serotype carriage** | | | **Comments** |
| --- | --- | --- | --- | --- | --- | --- | --- | --- |
|  |  |  |  |  | **Overall, %** | **PCV types, %** | **Non-PCV types, %** |  |
| [42] | Cheung et al. 2009 | The Gambia | PCV-9 or placebo | 9−15 mo (PCV-9) | 87.5 (RR=1.02; 0.98-1.05) | 22.6 (RR=0.56; 0.49-0.65) | 42.7 (RR=1.59; 1.41-1.79) | 9−15 mo (PCV-9):  - Serotypes 14, 19F, 6B, 23F, 4, and 9V less frequent.  - Serotypes 19A, 20, 34, 7C, 11, 15, 13, 22, 46, and 17 more frequent |
|  |  |  |  | 9−15 mo (placebo) | 86.2 | 40.0 | 26.9 |  |
|  |  |  |  | 21−27 mo (PCV-9) | 82.0 (RR=0.97; 0.93-1.01) | 25.0 (RR=0.61; 0.53-0.69) | 40.5 (RR=1.55; 1.35-1.77) | 21−27 mo (PCV-9):  - Serotypes 6B, 23F, 4, and 6A less frequent - Serotypes 21, 15, 10, 35B and 19A more frequent. |
|  |  |  |  | 21−27 mo (placebo) | 84.6 | 41.2 | 26.2 |  |
| [47] | Ota et al. 2011 | The Gambia | PCV-7  (1, 2, or 3 doses at 1, 2, and 3 mo) + PPV at 10 mo | 5 mo (1 dose) | 82.0 | 19.8 | 63.6 | The only significant difference between the 2-dose groups and the 3-dose groups was the carriage of PPV serotypes at 15 mo (42.9% vs. 32.0%, P=0.028). |
|  |  |  |  | 5 mo (2 dose) | 85.8 | 18.3 | 69.7 |  |
|  |  |  |  | 5 mo (3 dose) | 82.3 | 13.5 | 70.2 |  |
|  |  |  |  | 11 mo (1 dose) | 76.4 | 20.2 | 57.6 |  |
|  |  |  |  | 11 mo (2 dose) | 69.7 | 16.7 | 53.0 |  |
|  |  |  |  | 11 mo (3 dose) | 71.5 | 10.0 | 61.5 |  |
|  |  |  |  | 15 mo (1 dose) | 88.3 | 18.5 | 72.7 |  |
|  |  |  |  | 15 mo (2 dose) | 85.7 | 15.3 | 72.4 |  |
|  |  |  |  | 15 mo (3 dose) | 82.0 | 12.4 | 70.1 |  |
| [35] | Roca et al. 2012 | The Gambia | PCV-7; All village residents vaccinated vs. only children ≤30 mo vaccinated (control villages) | Randomly selected, age stratified residents (n = 1200) 4-6, 12, and 24 months after vaccination started | Pre-vaccination: 71 Post-vaccination: 44 |  |  | - No significant differences in post-vaccination carriage density between vaccinated and control villages for either vaccine-types or non-vaccine-types - In vaccinated villages, carriage density decreased significantly after vaccination in individuals >5 y [2.44 to 1.88, difference=−0.56, P=0.001]  - In control villages, carriage density decreased significantly after vaccination in individuals ≤ 5 y [2.76 to 1.99, Δ = −0.78, P=0.002]  and > 5 y [2.44 to 1.75, Δ = −0.69, P=0.001] |
| [53] | Roca et al. 2011 | The Gambia | PCV-7; All village residents vaccinated | 2 to < 5 y (pre) | 86.7 | 50.4 | 54.0 |  |
|  |  |  |  | 2 to <5 y (4−6 mo) | 87.8 | 20.0 | 67.8 |  |
|  |  |  |  | 2 to <5 y (12 mo) | 58.0 | 21.7 | 42.0 |  |
|  |  |  |  | 2 to <5 y (22 mo) | 76.7 | 13.3 | 63.3 |  |
|  |  |  |  | 5 to <15 y (pre) | 82.8 | 28.0 | 60.8 |  |
|  |  |  |  | 5 to <15 y (4−6 mo) | 61.3 | 5.9 | 57.4 |  |
|  |  |  |  | 5 to <15 y (12 mo) | 41.4 | 1.5 | 40.4 |  |
|  |  |  |  | 5 to <15 y (22 mo) | 68.9 | 6.1 | 62.9 |  |
|  |  |  |  | ≥15 y (pre) | 57.6 | 15.9 | 41.0 |  |
|  |  |  |  | ≥15 y (4−6 mo) | 31.1 | 4.2 | 27.8 |  |
|  |  |  |  | ≥15 y (12 mo) | 23.1 | 1.3 | 22.2 |  |
|  |  |  |  | ≥15 y (22 mo) | 29.2 | 0.0 | 29.2 |  |
|  |  |  | PCV-7; only children ≤ 30 mo vaccinated (control villages) | 2 to <5 y (pre) | 93.4 | 53.8 | 50.9 |  |
|  |  |  |  | 2 to <5 y (4−6 mo) | 89.8 | 28.8 | 66.1 |  |
|  |  |  |  | 2 to <5 y (12 mo) | 64.3 | 27.1 | 40.0 |  |
|  |  |  |  | 2 to <5 y (22 mo) | 78.9 | 23.7 | 60.5 |  |
|  |  |  |  | 5 to <15 y (pre) | 86.3 | 34.7 | 58.9 |  |
|  |  |  |  | 5 to <15 y (4−6 mo) | 63.0 | 8.7 | 56.3 |  |
|  |  |  |  | 5 to <15 y (12 mo) | 52.5 | 8.7 | 44.7 |  |
|  |  |  |  | 5 to <15 y (22 mo) | 66.7 | 14.8 | 52.8 |  |
|  |  |  |  | ≥15 y (pre) | 60.6 | 16.7 | 41.7 |  |
|  |  |  |  | ≥15 y (4−6 mo) | 24.2 | 4.4 | 20.5 |  |
|  |  |  |  | ≥15 y (12 mo) | 29.1 | 3.9 | 25.8 |  |
|  |  |  |  | ≥15 y (22 mo) | 34.8 | 7.6 | 30.3 |  |
| [46] | Akinsola et al. 2012 | The Gambia | PCV-9 followed by PCV-7 booster in 2−4 y children | 2−4 y (pre-booster) |  | 13 | 39 |  |
|  |  |  |  | 2−4 y (6−8 wk post-booster) |  | 11 | 39 |  |
|  |  |  |  | 2−4 y (5 mo post-booster) |  | 10 | 37 |  |
|  |  |  |  | 2−4 y (16−18 mo post booster) |  | 6 | 28 |  |
|  |  |  | Placebo followed by PCV-7 booster in 2−4 y children (control) | 2−4 y (pre-booster) |  | 17 | 28 |  |
|  |  |  |  | 2−4 y (6−8 wk post-booster) |  | 11 | 27 |  |
|  |  |  |  | 2−4 y (5 mo post-booster) |  | 9 | 38 |  |
|  |  |  |  | 2−4 y (16−18 mo post booster) |  | 2 | 26 |  |

NR, not reported; PCV-7, 7-valent pneumococcal conjugate vaccine; PCV-9, 9-valent pneumococcal conjugate vaccine; PPV, pneumococcal polysaccharide vaccine; mo, months; wk, week; y, years.
