# Supplementary material for: Carriage of Streptococcus pneumoniae and Other Respiratory Bacterial Pathogens in Low and Lower-Middle Income Countries: A Systematic Review and Meta-Analysis
Source: PLoS One. 2014 Aug 1;9(8):e103293. doi: 10.1371/journal.pone.0103293 (PMC4118866; doi:10.1371/journal.pone.0103293)
Supplement: Text S1 — Supplementary methods. (DOCX) [file pone.0103293.s009.docx]

**Text S1.** Supplementary methods

**Geographical scope of the review, as defined by the World Bank**

**Low income countries (gross national income per capita, $1,025 or less)**

Afghanistan, Bangladesh, Benin, Burkina Faso, Burundi, Cambodia, Central African Republic, Chad, Comoros, Democratic Republic of Congo, Eritrea, Ethiopia, The Gambia, Guinea, Guinea-Bissau, Haiti, Kenya, Democratic Republic of Korea, Kyrgyz Republic, Liberia, Madagascar, Malawi, Mali, Mauritania, Mozambique, Myanmar, Nepal, Niger, Rwanda, Sierra Leone, Somalia, Tajikistan, Tanzania, Togo, Uganda, Zimbabwe.

**Lower-middle income countries (gross national income per capita, $1,026 to $4,035)**

Albania, Armenia, Belize, Bhutan, Bolivia, Cameroon, Cape Verde, Republic Congo, Côte d'Ivoire, Djibouti, Arab Republic of Egypt, El Salvador, Fiji, Georgia, Ghana, Guatemala, Guyana, Honduras, Indonesia, India, Iraq, Kiribati, Kosovo, Lao People's Democratic Republic, Lesotho, Marshall Islands, Federated States of Micronesia, Moldova, Mongolia, Morocco, Nicaragua, Nigeria, Pakistan, Papua New Guinea, Paraguay, Philippines, Samoa, São Tomé and Principe, Senegal, Solomon Islands, South Sudan, Sri Lanka, Sudan, Swaziland, Syrian Arab Republic, Timor-Leste, Tonga, Ukraine, Uzbekistan, Vanuatu, Vietnam, West Bank and Gaza, Republic of Yemen, Zambia.

**Search strategy in PubMed (<http://www.ncbi.nlm.nih.gov/pubmed>)**

**Search string #1 for the pathogens:**

Respiratory bacterial pathogen*[tw] OR "Streptococcus pneumoniae"[Mesh] OR streptococcus pneumonia*[tw] OR S. pneumonia*[tw] OR pneumococcal[tw] OR "Haemophilus influenzae"[Mesh] OR haemophilus influenza*[tw] OR *H. influenzae**[tw] OR NTHi[tw] OR Hib[tw] OR hemophilus[tw] OR haemophilus[tw] OR "Moraxella (Branhamella) catarrhalis"[Mesh] OR moraxella catarrhalis[tw] OR Branhamella catarrhalis[tw] OR *M. catarrhalis*[tw] OR B. catarrhalis[tw] OR "Staphylococcus aureus"[Mesh] OR staphylococcus aureus[tw] OR *S. aureus*[tw] OR staphylococcal[tw] OR “Neisseria meningitidis”[Mesh] OR Neisseria meningitidis[tw] OR *N. meningitidis*[tw]

**Search string #2 for colonization and carriage:**

"carrier state"[mesh] OR carriage[tw] OR "Nose/microbiology"[Mesh] OR "pharynx/microbiology"[Mesh] OR "Nasopharynx/microbiology"[Mesh] OR "Oropharynx/microbiology"[Mesh] OR colonization[tiab] OR colonisation[tiab] OR colonizing[tiab] OR colonising[tiab] OR co-colonization[tiab] OR co-colonisation[tiab] OR multicolonization[tiab] OR multicolonisation[tiab] OR nasal bacterial load[tiab]

**Search string #3 for lower income countries:**

Afghanistan*[tw] OR Afghanistan*[ad] OR Gambia[tw] OR Gambian[tw] OR Gambia[ad] OR Gambian[ad] OR Mozambiqu*[tw] OR Mozambiqu*[ad] OR Banglades*[tw] OR Banglades*[ad] OR “Guinea-Bissau”[tw] OR “Guinea Bissau”[tw] OR “Guinea-Bissau”[ad] OR “Guinea Bissau”[ad] OR Myanmar[tw] OR Myanmar[ad] OR Birma[tw] OR Birma[ad] OR Benin*[tw] OR Benin*[ad] OR Nepal[tw] OR Nepal[ad] OR “Burkina Faso”[tw] OR “Burkina Faso”[ad] OR Haiti[tw] OR Haiti[ad] OR Niger[tw] OR Niger[ad] OR Burund*[tw] OR Burund*[ad] OR Keny*[tw] OR Keny*[ad] OR Rwand*[tw] OR Rwand*[ad] OR Cambod*[tw] OR Cambod*[ad] OR Kea[tw] OR Kea[ad] OR “Sierra Leone”[tw] OR “Sierra Leone”[ad] OR “Republique Centrafricaine”[tw] OR Centrafrique[tw] OR “Central African Republic”[tw] OR “Republique Centrafricaine”[ad] OR Centrafrique[ad] OR “Central African Republic”[ad] OR Kyrgyzstan*[tw] OR Kyrgyzstan*[ad] OR “Kyrgyz Republic”[tw] OR “Kyrgyz Republic”[ad] OR Somali*[tw] OR Somali*[ad] OR Tchad*[tw] OR Tchad*[ad] OR Chad*[tw] OR Chad*[ad] OR Liberia*[tw] OR Liberia*[ad] OR Tadjikistan*[tw] OR Tadjikistan*[ad] OR Comoros[tw] OR Comoros[ad] OR Madagascar*[tw] OR Madagascar*[ad] OR Tanzani*[tw] OR Tanzani*[ad] OR Congo*[tw] OR Congo*[ad] OR Malawi*[tw] OR Malawi*[ad] OR Togo[tw] OR Togolese[tw] OR Togo[ad] OR Togolese[ad] OR Erythrée[tw] OR Eritrea[tw] OR Erythrée[ad] OR Eritrea[ad] OR Mali[tw] OR Malian[tw] OR Mali[ad] OR Malian[ad] OR Ugand*[tw] OR Ugand*[ad] OR Ethiopi*[tw] OR Ethiopi*[ad] OR Mauritani*[tw] OR Mauritani*[ad] OR Zimbabw*[tw] OR Zimbabw*[ad]

**Search string #4 for lower-middle income countries:**

Albani*[tw] OR Albani*[ad] OR Indones*[tw] OR Indones*[ad] OR Samoa[tw] OR Samoa[ad] OR Armenia*[tw] OR Armenia*[ad] OR India[tw] OR Indian[tw] OR India[ad] OR Indian[ad] OR “Sao Tome and Principe”[tw] OR “São Tomé e Príncipe”[tw] OR “Sao Tome and Principe”[ad] OR “São Tomé e Príncipe”[ad] OR Belize[tw] OR Belize[ad] OR Iraq*[tw] OR Iraq*[ad] OR Senegal*[tw] OR Senegal*[ad] OR Bhutan[tw] OR Bhutan[ad] OR Kirbati[tw] OR Kirbati[ad] OR Solomon Island*[tw] OR Solomon Island*[ad] OR Bolivia[tw] OR Bolivia[ad] OR Kosovo[tw] OR Kosovo[ad] OR Cameroon*[tw] OR Cameroon*[ad] OR Lao*[tw] OR Lao*[ad] OR “Sri Lanka”[tw] OR “Sri Lanka”[ad] OR “Cape Verde”[tw] OR “Cape Verde”[ad] OR Sudan*[tw] OR Sudan*[ad] OR Lesotho*[tw] OR Lesotho*[ad] OR Congo*[tw] OR Congo*[ad] OR Marshall Island*[tw] OR Marshall Island*[ad] OR Swazi*[tw] OR Swazi*[ad] OR “Cote D'Ivoire”[tw] OR “Ivory Coast”[tw] OR “Cote D'Ivoire”[ad] OR “Ivory Coast”[ad] OR micronesi*[tw] OR micronesi*[ad] OR Syria*[tw] OR Syria*[ad] OR Djibout*[tw] OR Djibout*[ad] OR Moldov*[tw] OR Moldov*[ad] OR Timor-Leste[tw] OR Timor-Leste[ad] OR Egypt*[tw] OR Egypt*[ad] OR Mongolia*[tw] OR Mongolia*[ad] OR Tonga[tw] OT Tonga[ad] OR El Salvador[tw] OR El Salvador[ad] OR Morocc*[tw] OR Morocc*[ad] OR Ukrain*[tw] OR Ukrain*[ad] OR Fiji[tw] OR Fiji[ad] OR Nicaragua[tw] OR Nicaragua[ad] OR Ouzbekistan*[tw] OR Uzbekistan*[tw] OR Ouzbekistan*[ad] OR Uzbekistan*[ad] OR Georgi*[tw] OR Georgi*[ad] OR Nigeria*[tw] OR Nigeria*[ad] OR Vanuatu*[tw] OR Vanuatu*[ad] OR Ghana*[tw] OR Ghana*[ad] OR Pakistan*[tw] OR Pakistan*[ad] OR Vietnam*[tw] OR Vietnam*[ad] OR Guatamala[tw] OR Guatamala[ad] OR Papua New Guinea[tw] OR Papua New Guinea[ad] OR West Bank[tw] OR West Bank[ad] OR Gaza[tw] OR Gaza[ad] OR Gyan*[tw] OR Gyan*[ad] OR Paraguay[tw] OR Paraguay[ad] OR Yemen*[tw] OR Yemen*[ad] OR Honduras[tw] OR Honduras[ad] OR Philippine*[tw] OR Philippine*[ad] OR Zambia*[tw] OR Zambia*[ad]

The asterisk (*) represents any group of characters (including no character). This truncation allows PubMed to find all terms that begin with a given text string.

**Limits:**

Publication date: 1990/01/01 to present. The searched was performed on October, 23^rd^ 2012

Language: English

**Search string combinations**

#1 AND #2 AND (#3 OR #4) + limits

**Search strategy in additional websites (grey literature)**

- World Health Organization (WHO): www.who.int

- United Nations International Children's Emergency Fund (UNICEF): www.unicef.org

- Google: country name + *S. pneumoniae*, *H. influenzae*, *M. catarrhalis*, *S. aureus,* or *N. meningitidis* + carriage
