# Supplementary material for: Carriage of Streptococcus pneumoniae and Other Respiratory Bacterial Pathogens in Low and Lower-Middle Income Countries: A Systematic Review and Meta-Analysis
Source: PLoS One. 2014 Aug 1;9(8):e103293. doi: 10.1371/journal.pone.0103293 (PMC4118866; doi:10.1371/journal.pone.0103293)
Supplement: Protocol S1 — Protocol of the systematic review. (PDF) [file pone.0103293.s010.pdf]

Systematic review

Nasopharyngeal and oropharyngeal carriage of *S. Pneumoniae, H. Influenzae, M. Catarrhalis, N. Meningitidis and S. Aureus* in low and lower middle income countries

1 Background and objectives

1.1 Background

Introduction of pneumococcal conjugate vaccines (P CVs) into the routine infant immunisation programmes of industrialised countries has had a major impact on invasive pneumococcal disease in countries where this has been done. A remarkable feature of PCVs has been their impact on non-vaccinated older subjects and it has been estimated that in the USA the number of cases of invasive pneumococcal disease (IPD) prevented through their indirect herd effect has been higher than the number of cases prevented in vaccinated children. It is likely that this high degree of indirect protection has been achieved because in industrialised countries most transmission of pneumococci is from young children to their parents and grandparents. Thus, prevention of carriage in infants and young children has provided a high level of indirect protection to their older contacts.

Pneumococcal conjugate vaccines are being now introduced rapidly into the routine infant immunisation (EPI) programme of developing countries including some of the highest risk countries in Africa, with support from GAVI, because of the high burden of IPD in young children in the developing world. Although there are only limited data it is likely that IPD is also an important problem in adults in many developing countries in particular those with a high incidence of HIV. As average life time increase in developing countries the proportion of the population who have risk factors for IPD such as diabetes and chronic respiratory infections is increasing and it is hoped that a similar indirect effect of vaccinating infants will be seen in these at risk populations as seen in the USA and Europe.

However, this will only happen if the major route of transmission of pneumococci in developing countries is from is from young children to adults and it is not certain whether this is the case. Because the overall carriage of rate in all age groups is much higher than in industrialised countries and the risk factors for carriage different from those seen in industrialised countries adult to adult transmission may be much more important.

.

1.2 Business objectives

GSK Biologicals would like to have a systematic review of published and publicly available data on the nasopharyngeal and oropharyngeal carriages rates of S. pneumoniae, H. influenzae, M. catarrhalis, S. aureus and N. meningitides in children and adults and the associated risk factors in lower income countries and lower middle income countries. Furthermore, GSK Biologicals is interested in:

- the association between carriage and disease (acute otitis media, invasive pneumococcal disease and sinusitis and respiratory tract infection) with and without regard to time;
- the evidence on carriage as a prerequisite for the development of the above mentioned diseases;
- most common factors that lead from carriage to disease;
- impact of vaccination on carriage in vaccinated subjects as well as non-vaccinated subjects;
- serotype and pathogen distribution of carriage and its geographical differences.

1.3 Review objectives

Based on the background situation and business objectives of GSK Biologicals, we have phrased the following review questions:

1. What are nasopharyngeal and oropharyngeal carriages rates of the above mentioned microbiological agents in children and adults?
2. What are the risk factors and their estimates for nasopharyngeal and oropharyngeal carriage of the above mentioned agents in children and adults?
3. What is the association, without regard to time, between carriage of the above-mentioned microbiological agents and acute otitis media, invasive pneumococcal disease, sinusitis and respiratory tract infections?
4. What is the evidence for carriage as a prerequisite for the development of acute otitis media, invasive pneumococcal disease and respiratory tract infections?
5. What are the most common factors that lead from carriage into disease (acute otitis media, invasive pneumococcal disease, sinusitis and respiratory tract infections)?
6. What is the serotype and pathogen distribution of carriage and do geographical differences exist?
7. What is the impact of vaccination on carriage, including serotype distribution (vaccinated vs. non-vaccinated as well as pre-vaccination vs. post-vaccination).

1.4 Variables of particular importance

GSK defined the following variables of particular importance:

<u>Risk factors</u>

- Having (older) siblings
  - Only 1
  - >1
  - >3
  - >5
- Day-care (pre-school) attendance
- Breast-feeding
- Use of pacifier
- Living in a rural/urban area
- Living in crowded conditions
- Age
  - Neonatal (pre-term)
  - <1
  - >1
  - 2-3
  - >5
- Socio-economic status (low, medium and high)
- Genetic
- Vaccination status (PCV, Hib, ect.)
- Medical history
- Underlying medical conditions (preceding or simultaneous viral infection, respiratory illness, stroke, diabetes, etc.)
- Immunocompromised status (HIV, treatment, sickle cell, asplenic, malaria, etc.)
- Recent antibiotic treatment, recent steroid treatment
- Born pre-term
- Co-colonization / isolation other viral/bacterial pathogen
- Seasonality
- Environmental exposure
- Air pollution
- Indoor pollution
- Exposure to tobacco smoke in the house

<u>Other variables:</u>

- Age specific and time trends of pathogen and serotype specific *S. pneumoniae, N. meningitides, S. aureus, H. influenzae* and *M.* catharralis carriage rates stratified by country/region.
- Racial/ethnic differences in carriage rates (and disease if possible)

Pallas will include these variables in the evidence tables.

1.5 Geographical scope

The geographical scope is low and lower middle income countries.

- Low income countries: Afghanistan, Gambia, Mozambique, Bangladesh, Guinea, Myanmar, Benin, Nepal, Burkina, Haiti, Niger, Burundi, Kenya, Rwanda, Cambodia, Kea, Sierra Leone, Central African Republic, Kyrgyz Republic, Somalia, Chad, Liberia, Tajikistan, Comoros, Madagascar, Tanzania, Congo, Malawi, Togo, Eritrea, Mali, Uganda, Ethiopia, Mauritania, Zimbabwe.

- Lower middle income countries: Albania, Indonesia, Samoa, Armenia, India, Sao Tome and Principe", Belize, Iraq, Senegal, Bhutan, Kiribati, Solomon Islands, Bolivia, Kosovo, South Sudan, Cameroon, Lao, Sri Lanka, Cape Verde, Lesotho, Sudan, Congo, Marshall Islands, Swaziland, Ivory Coast, Micronesia, Syrian Arab Republic, Djibouti, Moldova, Timor-Leste, Egypt, Mongolia, Tonga, El Salvador, Morocco, Ukraine, Fiji, Nicaragua, Uzbekistan, Georgia, Nigeria, Vanuatu, Ghana, Pakistan, Vietnam, Guatemala, Papua New Guinea, West Bank and Gaza, Guyana, Paraguay, Yemen, Honduras, Philippines, Zambia.

2 Proposed review method

In order to meet the review objectives, as outlined in section 1.3, Pallas will perform a systematic review of the literature. A systematic review is a method to collect, critically appraise and summarize the best available evidence in a transparent and systematic way using generally accepted evidence-based principles.

Pallas will finalize the search strategies, select literature based on title and abstracts, and will critically appraise full-text articles based on checklists for Evidence Based Medicine, and summarize the evidence in consultation with GSK Biologicals. Results will be documented in evidence tables and exclusion tables in order to ensure transparency and reproducibility of the results. The review steps are further outlined below.

2.1. Analysis international peer reviewed literature

The core of our review will be a PubMed literature search. We will conduct the search in PubMed as described below.

2.1.1 Search strings

Pallas made four search strings, one on microbiological agents, one on carriage and colonization, one on low income countries and one on lower middle income countries of interest. Within one search string, possible relevant search terms are combined using ‘OR’. To end up with a pool of articles possible relevant for this review, the results from search string 1 and 2 will be run in combination with search string 3 and 4 for the selected countries (i.e. #1 AND #2 AND (#3 OR #4).

The following search string will be used to identify the microbiological agents :
Respiratory bacterial pathogen*[tw] OR "Streptococcus pneumoniae"[Mesh] OR streptococcus pneumonia*[tw] OR S. pneumonia*[tw] OR pneumococcal[tw] OR "Haemophilus influenzae"[Mesh] OR haemophilus influenza*[tw] OR H. influenza*[tw] OR NTHi[tw] OR Hib[tw] OR hemophilus[tw] OR haemophilus[tw] OR "Moraxella (Branhamella) catarrhalis"[Mesh] OR moraxella catarrhalis[tw] OR Branhamella catarrhalis[tw] OR M. catarrhalis[tw] OR B. catarrhalis[tw] OR "Staphylococcus aureus"[Mesh] OR staphylococcus aureus[tw] OR S. aureus[tw] OR staphylococcal[tw] OR Neisseria meningitidis[mesh] OR Neisseria meningitidis[tw] OR N. meningitidis[tw]

The following string will be used for colonization and carriage:
"carrier state"[mesh] OR carriage[tw] OR "Nose/microbiology"[Mesh] OR "pharynx/microbiology"[Mesh] OR "Nasopharynx/microbiology"[Mesh] OR "Oropharynx/microbiology"[Mesh] OR colonization[tiab] OR colonisation[tiab] OR colonizing[tiab] OR colonising[tiab] OR co-colonization[tiab] OR co-colonisation[tiab] OR multicolonization[tiab] OR multicolonization[tiab] OR nasal bacterial load[tiab]

The following string will be used for lower income countries:
Afghanistan*[tw] OR Afghanistan*[ad] OR Gambia[tw] OR Gambian[tw] OR Gambia[ad] OR Gambian[ad] OR Mozambiqu*[tw] OR Mozambiqu*[ad] OR Banglades*[tw] OR Banglades*[ad] OR “Guinea-Bissau”[tw] OR “Guinea Bissau”[tw] OR “Guinea-Bissau”[ad] OR “Guinea Bissau”[ad] OR Myanmar[tw] OR Myanmae[ad] OR Birma[tw] OR Brima[ad] OR Benin*[tw] OR Benin*[ad] OR Nepal[tw] OR Nepal[ad] OR “Burkina Faso”[tw] OR “Burkina Faso”[ad] OR Haiti[tw] OR Haiti[ad] OR Niger[tw] OR Niger[ad] OR Burund*[tw] OR Burund*[ad] OR Keny*[tw] OR Keny*[ad] OR Rwand*[tw] OR Rwand*[ad] OR Cambod[tw] OR Cambod[ad] OR Kea[tw] OR Kea[tw] OR “Sierra Leone”[tw] OR “Sierra Leone”[ad] OR “Republique Centrafricaine”[tw] OR Centrafrique[tw] OR “Central African Republic”[tw] OR “Republique Centrafricaine”[ad] OR Centrafrique[ad] OR “Central African Republic”[ad] OR Kyrgyzstan*[tw] OR Kyrgyzstan*[ad] OR Kyrgyz republic[tw] OR Kyrgyz republic[ad] OR Somali*[tw] OR Somali*[ad] OR Tchad*[tw] OR Tchad*[ad] OR Chad*[tw] OR Chad*[ad] OR Liberia*[tw] OR Liberia*[ad]

OR Tadjikistan*[tw] OR Tadjikistan*[ad] OR Comoros[tw] OR Comoros[ad] OR Madagascar*[tw] OR Madagascar*[ad] OR Tanzani*[tw] OR Tanzani*[ad] OR Congo*[tw] OR Congo*[ad] OR Malawi*[tw] OR Malawi*[ad] OR Togo[tw] OR Togolese[tw] OR Togo[ad] OR Togolese[ad] OR Erythrée[tw] OR Eritrea[tw] OR Erythrée[ad] OR Eritrea[ad] OR Mali[tw] OR Malian[tw] OR Mali[ad] OR Malian[ad] OR Ugand*[tw] OR Ugand*[ad] OR Ethiopi*[tw] OR Ethiopi*[ad] OR Mauritani*[tw] OR Mauritani*[ad] OR Zimbabw*[tw] OR Zimbabw*[ad]

The following string will be used for lower middle income countries:
Albani*[tw] OR Albani*[ad] OR Indones*[tw] OR Indones*[ad] OR Samoa[tw] OR Samoa[ad] OR Armenia*[tw] OR Armenia*[ad] OR India[tw] OR Indian[tw] OR India[ad] OR Indian[ad] OR "Sao Tome and Principe"[tw] OR "São Tomé e Príncipe"[tw] OR "Sao Tome and Principe"[ad] OR "São Tomé e Príncipe"[ad] OR Belize[tw] OR Belize[ad] OR Iraq*[tw] OR Iraq*[ad] OR Senegal*[tw] OR Senegal*[ad] OR Bhutan[tw] OR Bhutan[ad] OR Kirbati[tw] OR Kirbati[ad] OR Solomon Island*[tw] OR Solomon Island*[ad] OR Bolivia[tw] OR Bolivia[ad] OR Kosovo[tw] OR Kosovo[ad] OR Sudan*[tw] OR Sudan*[ad] OR Cameroon*[tw] OR Cameroon*[ad] OR Lao*[tw] OR Lao*[ad] OR "Sri Lanka"[tw] OR "Sri Lanka"[ad] OR "Cape Verde"[tw] OR "Cape Verde"[ad] OR Sudan*[tw] OR Sudan*[ad] OR Lesotho*[tw] OR Lesotho*[ad] OR Congo*[tw] OR Congo*[ad] OR Marshall Island*[tw] OR Marshall Island*[ad] OR Swazi*[tw] OR Swazi*[ad] OR "Cote D'Ivoire"[tw] OR "Ivory Coast"[tw] OR "Cote D'Ivoire"[ad] OR "Ivory Coast"[ad] OR micronesi*[tw] OR micronesi*[ad] OR Syria*[tw] OR Syria*[ad] OR Djibout*[tw] OR Djibout*[ad] OR Moldov*[tw] OR Moldov*[ad] OR Timor-Leste[tw] OR Timor-Leste[ad] OR Egypt*[tw] OR Egypt*[ad] OR Mongolia*[tw] OR Mongolia*[ad] OR Tonga[tw] OT Tonga[ad] OR El Salvador[tw] OR El Salvador[ad] OR OR Morocc*[tw] OR Morocc*[ad] OR Ukrain*[tw] OR Ukrain*[ad] OR Fiji[tw] OR Fiji[ad] OR Nicaragua[tw] OR Nicaragua[ad] OR Ouzbekistan*[tw] OR Uzbekistan*[tw] OR Ouzbekistan*[ad] OR Uzbekistan*[ad] OR Georgi*[tw] OR Georgi*[ad] OR Nigeria*[tw] OR Nigeria*[ad] OR Vanuatu*[tw] OR Vanuatu*[ad] OR Ghana*[tw] OR Ghana*[ad] OR Pakistan*[tw] OR Pakistan*[ad] OR Vietnam*[tw] OR Vietnam*[ad] OR Guatamala[tw] OR Guatamala[ad] OR Papua New Guinea[tw] OR Papua New Guinea[ad] OR West Bank[tw] OR West Bank[ad] OR Gaza[tw] OR Gaza[ad] OR Gyan*[tw] OR Gyan*[ad] OR Paraguay[tw] OR Paraguay[ad] OR Yemen*[tw] OR Yemen*[ad] OR Honduras[tw] OR Honduras[ad] OR Philippine*[tw] OR Philippine*[ad] OR Zambia*[tw] OR Zambia*[tw]

Limits
The following limits will be applied:

- Publication date: 1990/01/01 to 2012/10/01
- Language: English

A combination of the above mentioned search strings and limits yields 324 unique articles (October 3, 2012). We will not further limit the search, e.g. on outcome measures, since the total number of hits on microbiological agents and colonization in the selected countries is low enough to be checked completely. In case insufficient data are available, it will be discussed with GSK whether searching for published articles before 1990 is relevant.

2.1.2 Selection procedure

From the articles retrieved from PubMed the relevant references will be selected by a three-step selection procedure, based on:

1. Screening of title and abstract (first selection step): this step yields the articles that will be assessed in full text.

   - The major topics of the articles will be assessed by the title and abstract. Articles that do not contain information relevant to the research objectives will not be selected for full text assessment. Articles that will be excluded are, for example, diagnostic test research, letters to the editor, editorials or comments.

2. Screening of full article (second selection step): in this step the full text articles, selected in step 1, will be assessed. These articles will either be included in the report or will be excluded when it turns out that the article does not contain relevant information or that the information is of poor quality.

- In this stage critical appraisal of full text articles using a standard set of criteria (see section 2.1.5) will take place.
- Examples of exclusion criteria in this stage include: a review not conducted in a systematic way, methods section not sufficiently described, etc.

3. Screening during data-extraction phase:
In case of meta-analysis or good quality systematic reviews, in which sufficient details of the original studies are described, the original articles of the meta-analysis/review will not be included separately.

The process of selection and in- and exclusion of articles will be registered in an Endnote library by one of the researchers. In this way, a clear overview on all selection steps will be maintained at all phases. The exclusion criteria applied in all steps of this selection procedure will be reported on in the methods-section of the report.

Inclusion and exclusion criteria

Inclusion criteria:

- Articles on children and adults
- Articles relevant for the review objectives

Exclusion criteria:

- Data from letters, editorials or comments.
- Articles with lacking methodology (e.g. not systematic reviews, bias).
- Diagnostic articles

2.1.3 Study quality assessment checklists and procedures

The Pallas team will critically appraise the methodological quality of the articles. Pallas will use the Coordination of Cancer Clinical Practice Guidelines (CoCanCPG) checklists for different study designs to do so. The CoCanCPG was originally designed for developing cancer guidelines, but the criteria are also applicable to studies that address other research questions.
For this particular project, the majority of the study types will probably concern cross-sectional or longitudinal studies. There are no standard checklists for these types available. For the GSK review project on carriage rates of *S. pneumoniae, H. influenzae, M. catarrhalis* and *S. aureus* in Europe and Northern America, we used the CoCanCPG checklist for cohort studies as a basis to assess cross-sectional studies, and added a few other criteria applicable for the purpose of that review, e.g. GSK needed the study method and typing method clearly described. See example below; Pallas' additions to the CoCanCPG checklist for cohort studies are in italic.

- The study addresses an appropriate and clearly focused question.
- The outcomes are clearly defined
- Are the results of the cohort applicable to the patient group targeted in the search question?

Additional questions:

- *The population is a representative sample of the source population*
- *Study design and setting is described*
- *The type of sample for aetiology is described*
- *The typing method is described*

2.1.4 Data extraction

Pallas proposes to create 3 separate evidence tables.

- Table 1: objective 1 and 2
- Table 2: objective 3, 4 and 5
- Table 3: objective 6 and 7

Table 1 and 2 will be tables in Word. Table 3 will be put in Excel format. The results will be presented for children and adults separately.

In case an article presents relevant figures that cannot be incorporated in evidence tables, the figure will be put in the main text of the results section of the report provided with a short explanation and the reference.

Some of the research questions may overlap in the literature. One researcher will carry out full data-extraction for one article, also when this article is relevant for multiple objectives.

2.1.5 Quality control

The following quality control measures will be put in place:

- The first 30% of titles and abstracts will be screened in duplicate by two independent researchers from Pallas. The results will be compared and discussed before the remaining references are assessed by one researcher.
- The first 10% of full text articles will be critically appraised in duplicate by two independent researchers from Pallas. The results will be compared and discussed early in the process. Any disagreements will be adjudicated by a third researcher if necessary.
- Data extraction: the evidence tables will be compiled by junior researchers and reviewed by the senior researcher of the project.

2.1.6 Reproducibility

Pallas will document the review process in a way that assures reproducibility of the results. This includes documentation of final search strings and date of search, and documentation of the selection procedure.

2.2 Grey literature and other data sources

An additional search in grey literature and other data-sources will be conducted wherever important data seems missing.
The following websites are proposed for a grey data search:

- World Health Organisation (WHO): www.who.int
- UNICEF; www.unicef.org
- Ministries of health from most important countries (to be determined in collaboration with GSK)
- Google search (country name and carriage of one of the microbiological agents, most important countries to be determined with GSK)

2.3 Reference listings of key references

Where relevant, listings of key references will be checked for relevant other articles.

2.4 Analysing gaps in information

Gaps in information will arise from the identification of the available evidence from the peer-reviewed literature. Gaps in information will be reported by geographic region. Gaps in information will not only include review objectives with no evidence at all, but also objectives with lack of recent information, or lack of high level evidence. In the discussion section of the report we will comment on the gaps in information and prioritize those.

2.5 Presentation of the results

2.5.1 Evidence tables

Included articles will be summarized using data-extraction tables. Evidence tables of one article will be presented on a single page. The articles will be sorted by objective and per objective by region/country and study period.

The tables will include:

- First author and main institutions, journal, year of publication
- Country, study design and study period
- Setting and study population (including sample size)
- Type and number of samples for aetiology
- Typing method
- Type of agent
- Outcome measure (carriage rate, risk factors, association carriage and disease and risk factors, time span between carriage and onset disease, serotype distribution etc.)
- Comments

During project kick-off, the format of the evidence tables will be further refined in discussion with GSK Biologicals.

2.5.2 Summary tables

Key findings will be summarized in concise tables, the lay-out of which will be discussed with GSK.

2.6 Deliverables

2.6.1 Report

Pallas will deliver a full report answering the research questions and providing gaps in the literature.

The outline of the final report is proposed as follows:

- Summary
- Contents
- Background and objectives
- Methods:
  - Search strategy
  - In-/exclusion criteria
  - Critical appraisal
- Results:
  - Flow chart (nr. of total hits, in and excluded articles per selection step)
  - Key findings in summary tables and text
- Discussion:
  - Discussion of results
  - Gaps in information
  - Conclusions
- Appendix:
  - Evidence tables
- Abbreviations used
- List of references

2.6.2 Citation database

Pallas will deliver a citation database of identified references. Pallas prefers to work with Endnote.

2.7 Process management

Pallas will appoint one of its senior staff to be the day-to-day contact for GSK Biologicals for this project. At the start of, and during the project, she will contact her GSK Biologicals counterpart to further discuss work plan, scope and background of the project, and presentation of the results.

- Kick-off meeting, a face-to-face or TC with GSK's designated epidemiologists to finalize, e.g.:
  - objectives/data points of interest
  - lay-out evidence tables
  - timelines

- Weekly/bi-weekly phone-updates to discuss progress and to be used among other things to (if necessary)
  - Share a selection of articles in data-extraction table with GSK-epidemiologists to gather their comments e.g. regarding level of detail.
  - Discuss next steps.
  - Discuss (outline of) draft report(s).

PRISMA 2009 Checklist

Section/topic	#	Checklist item	Reported on page #
**TITLE**			
Title	1	Identify the report as a systematic review, meta-analysis, or both.	
**ABSTRACT**			
Structured summary	2	Provide a structured summary including, as applicable: background; objectives; data sources; study eligibility criteria, participants, and interventions; study appraisal and synthesis methods; results; limitations; conclusions and implications of key findings; systematic review registration number.	
**INTRODUCTION**			
Rationale	3	Describe the rationale for the review in the context of what is already known.	
Objectives	4	Provide an explicit statement of questions being addressed with reference to participants, interventions, comparisons, outcomes, and study design (PICOS).	
**METHODS**			
Protocol and registration	5	Indicate if a review protocol exists, if and where it can be accessed (e.g., Web address), and, if available, provide registration information including registration number.	
Eligibility criteria	6	Specify study characteristics (e.g., PICOS, length of follow-up) and report characteristics (e.g., years considered, language, publication status) used as criteria for eligibility, giving rationale.	
Information sources	7	Describe all information sources (e.g., databases with dates of coverage, contact with study authors to identify additional studies) in the search and date last searched.	
Search	8	Present full electronic search strategy for at least one database, including any limits used, such that it could be repeated.	

Study selection	9	State the process for selecting studies (i.e., screening, eligibility, included in systematic review, and, if applicable, included in the meta-analysis).	
Data collection process	10	Describe method of data extraction from reports (e.g., piloted forms, independently, in duplicate) and any processes for obtaining and confirming data from investigators.	
Data items	11	List and define all variables for which data were sought (e.g., PICOS, funding sources) and any assumptions and simplifications made.	
Risk of bias in individual studies	12	Describe methods used for assessing risk of bias of individual studies (including specification of whether this was done at the study or outcome level), and how this information is to be used in any data synthesis.	
Summary measures	13	State the principal summary measures (e.g., risk ratio, difference in means).	
Synthesis of results	14	Describe the methods of handling data and combining results of studies, if done, including measures of consistency (e.g., I^2) for each meta-analysis.	
Risk of bias across studies	15	Specify any assessment of risk of bias that may affect the cumulative evidence (e.g., publication bias, selective reporting within studies).	
Additional analyses	16	Describe methods of additional analyses (e.g., sensitivity or subgroup analyses, meta-regression), if done, indicating which were pre-specified.	
**RESULTS**			
Study selection	17	Give numbers of studies screened, assessed for eligibility, and included in the review, with reasons for exclusions at each stage, ideally with a flow diagram.	
Study characteristics	18	For each study, present characteristics for which data were extracted (e.g., study size, PICOS, follow-up period) and provide the citations.	
Risk of bias within studies	19	Present data on risk of bias of each study and, if available, any outcome level assessment (see item 12).	
Results of individual studies	20	For all outcomes considered (benefits or harms), present, for each study: (a) simple summary data for each intervention group (b) effect estimates and confidence intervals, ideally with a forest plot.	
Synthesis of results	21	Present results of each meta-analysis done, including confidence intervals and measures of consistency.	
Risk of bias across studies	22	Present results of any assessment of risk of bias across studies (see Item 15).	
Additional analysis	23	Give results of additional analyses, if done (e.g., sensitivity or subgroup analyses, meta-regression [see Item 16]).	

**DISCUSSION**			
Summary of evidence	24	Summarize the main findings including the strength of evidence for each main outcome; consider their relevance to key groups (e.g., healthcare providers, users, and policy makers).	
Limitations	25	Discuss limitations at study and outcome level (e.g., risk of bias), and at review-level (e.g., incomplete retrieval of identified research, reporting bias).	
Conclusions	26	Provide a general interpretation of the results in the context of other evidence, and implications for future research.	
**FUNDING**			
Funding	27	Describe sources of funding for the systematic review and other support (e.g., supply of data); role of funders for the systematic review.	

From: Moher D, Liberati A, Tetzlaff J, Altman DG, The PRISMA Group (2009). Preferred Reporting Items for Systematic Reviews and Meta-Analyses: The PRISMA Statement. PLoS Med 6(6): e1000097. doi:10.1371/journal.pmed1000097
